# miR-17 deregulates a core RUNX1-miRNA mechanism of CBF acute myeloid leukemia

**DOI:** 10.1186/s12943-014-0283-z

**Published:** 2015-01-23

**Authors:** John Adams Fischer, Stefano Rossetti, Arani Datta, Kevin Hasegawa Eng, Alessandro Beghini, Nicoletta Sacchi

**Affiliations:** Department of Cancer Genetics, Roswell Park Cancer Institute, Buffalo, NY 14263 USA; Department of Biostatistics and Bioinformatics, Roswell Park Cancer Institute, Buffalo, NY 14263 USA; Department of Medical Biotechnology and Translational Medicine, University of Milan, Milan, 20133 Italy

**Keywords:** Core Binding Factor, Acute myeloid leukemia, RUNX1, microRNAs, KIT, Proliferation, Myeloid differentiation

## Abstract

**Background:**

Core Binding Factor acute myeloid leukemia (CBF-AML) with t(8;21) RUNX1-MTG8 or inv(16) CBFB-MYH11 fusion proteins often show upregulation of wild type or mutated KIT receptor. However, also non-CBF-AML frequently displays upregulated KIT expression. In the first part of this study we show that KIT expression can be also upregulated by miR-17, a regulator of RUNX1, the gene encoding a CBF subunit. Interestingly, both CBF leukemia fusion proteins and miR-17, which targets RUNX1-3′UTR, negatively affect a common core RUNX1-miRNA mechanism that forces myeloid cells into an undifferentiated, KIT-induced, proliferating state. In the second part of this study we took advantage of the conservation of the core RUNX1-miRNA mechanism in mouse and human, to mechanistically demonstrate in a mouse myeloid cell model that increased KIT-induced proliferation is *per se* a mechanism sufficient to delay myeloid differentiation.

**Methods:**

Human (U937) or mouse (32D) myeloid clonal lines were used, respectively, to test: 1) the effect of RUNX1-MTG8 and CBFB-MYH11 fusion proteins, or upregulation of miR-17, on KIT-induced proliferation and myeloid differentiation, and 2) the effect of upregulation of KIT-induced proliferation *per se* on myeloid cell differentiation.

**Results:**

In the first part of this study we found that stable miR-17 upregulation affects, like the CBF-AML fusion proteins (RUNX1-MTG8 or CBFB-MYH11), a core RUNX1-miRNA mechanism leading to KIT-induced proliferation of differentiation-arrested U937 myeloid cells. In the second part of the study we harnessed the conservation of this core mechanism in human and mouse to demonstrate that the extent of KIT upregulation in 32D mouse myeloid cells with wild type RUNX1 can *per se* delay G-CSF-induced differentiation. The integrated information gathered from the two myeloid cell models shows that RUNX1 regulates myeloid differentiation not only by direct transcriptional regulation of coding and non-coding myeloid differentiation functions (e.g. miR-223), but also by modulating KIT-induced proliferation via non-coding miRNAs (e.g. miR-221).

**Conclusions:**

The novelty of this study is dual. On the one hand, miRNAs (e.g. miR-17) can mimic the effects of CBF-AML fusion proteins by affecting a core RUNX1-miRNA mechanism of KIT-induced proliferation of undifferentiated myeloid cells. On the other hand, the extent of KIT-induced proliferation itself can modulate myeloid differentiation of cells with wild type RUNX1 function.

**Electronic supplementary material:**

The online version of this article (doi:10.1186/s12943-014-0283-z) contains supplementary material, which is available to authorized users.

## Background

The core binding factor (CBF), a master hematopoietic transcription factor that controls the transcription of genes involved in both embryonic and post-embryonic hematopoietic development, is composed of two subunits: the CBFA2 subunit, encoded by the RUNX1 (AML1) gene on chromosome 21, and the CBFB subunit, encoded by the CBFB gene on chromosome 16. While RUNX1 binds DNA at specific consensus sequences in the regulatory regions of target genes, CBFB strengthens RUNX1 DNA-binding [1-4]. Both RUNX1 and CBFB undergo chromosomal rearrangements in acute myeloid leukemia (AML). The two most common AML chromosome rearrangements, the t(8;21) (q22; q22) and the inv(16) (p13; q22), generate the RUNX1-MTG8 (AML1-ETO) and CBFB-MYH11 fusion proteins, respectively. According to the French-American-British (FAB) classification, t(8;21)-positive leukemia is mostly M2, while inv(16)-positive leukemia is mostly M4Eo [3,5-7].

RUNX1-MTG8 not only affects RUNX1 allelic dosage, but also exerts a dominant negative action on RUNX1 target genes [1,5]. CBFB-MYH11, by binding RUNX1, also exerts a dominant negative action on RUNX1 target genes and, by sequestering RUNX1 into the cytoplasm, depletes RUNX1 in the nucleus [5,8,9]. Thus, even if in a different manner, both CBF-AML fusion proteins affect RUNX1 dosage as well as RUNX1 transcriptional regulatory function of both coding and non-coding RUNX1-target genes implicated in hematopoiesis [5,8,10,11].

The first described non-coding RUNX1-target was miR-223, which is critical for the establishment of both granulocyte and monocyte lineages [12-15]. RUNX1-MTG8 binding to RUNX1 consensus sequences in the miR-223 promoter region induces miR-223 transcriptional repression and block of myeloid differentiation in t(8;21) leukemia cells [12]. Subsequently, we found that the miR-222/221 gene cluster, involved in the regulation of the KIT receptor by targeting KIT-3'UTR [16], is also a direct RUNX1-target, whose transcription is repressed by the dominant negative t(8;21) RUNX1-MTG8 fusion protein [17].

We also reported that both t(8;21) and inv(16) CBF-AML display lower levels of miR-221 and miR-222 relative to non-CBF-AML, in association with increased expression of the KIT receptor (CD117 antigen) [17]. In this study we mechanistically demonstrate that, like RUNX1-MTG8, also CBFB-MYH11 interferes with RUNX1-CBFB. Thus, both CBF-AML fusion proteins negatively affect both the RUNX1-miR-221-KIT axis and RUNX1-miR-223 transcription, leading to increased KIT-induced proliferation of undifferentiated myeloid cells.

Interestingly, a significant number of non-CBF-AMLs also display elevated levels of KIT relative to non-leukemic cells [18-21]. Intrigued by this observation, we tested whether other factors, capable of interfering with RUNX1-CBFB regulatory function, can also negatively affect the RUNX1-miR-221-KIT axis and miR-223 transcription. In this study we focused on miR-17, a miRNA that, by targeting RUNX1-3′UTR, plays a key role in the control of RUNX1 expression and myeloid differentiation [22]. We found that the effects of ectopic miR-17 expression mimic the biological effects induced by the RUNX1-MTG8 and CBFB-MYH11 fusion proteins by affecting the same core mechanism: the RUNX1-miR-221-KIT axis and miR-223.

In the second part of this study we took advantage of the core mechanism conservation between human and mouse so that we could use the 32D mouse myeloid cell model to monitor cytokine-induced myeloid differentiation over a period of 12 days. In the 32D model, with a functional wild type RUNX1, we could demonstrate that in the absence of any factor that negatively interferes with RUNX1, the extent of KIT-induced proliferation *per se* determines a delay of cytokine-induced myeloid differentiation. Thus, KIT-induced proliferation is a mechanism that normally determines the timing of RUNX1-mediated myeloid differentiation processes.

## Results

### Both t(8;21) and inv(16) leukemia fusion proteins affect the same RUNX1-miRNA-KIT axis regulating KIT proliferation activity

**Figure 1:**
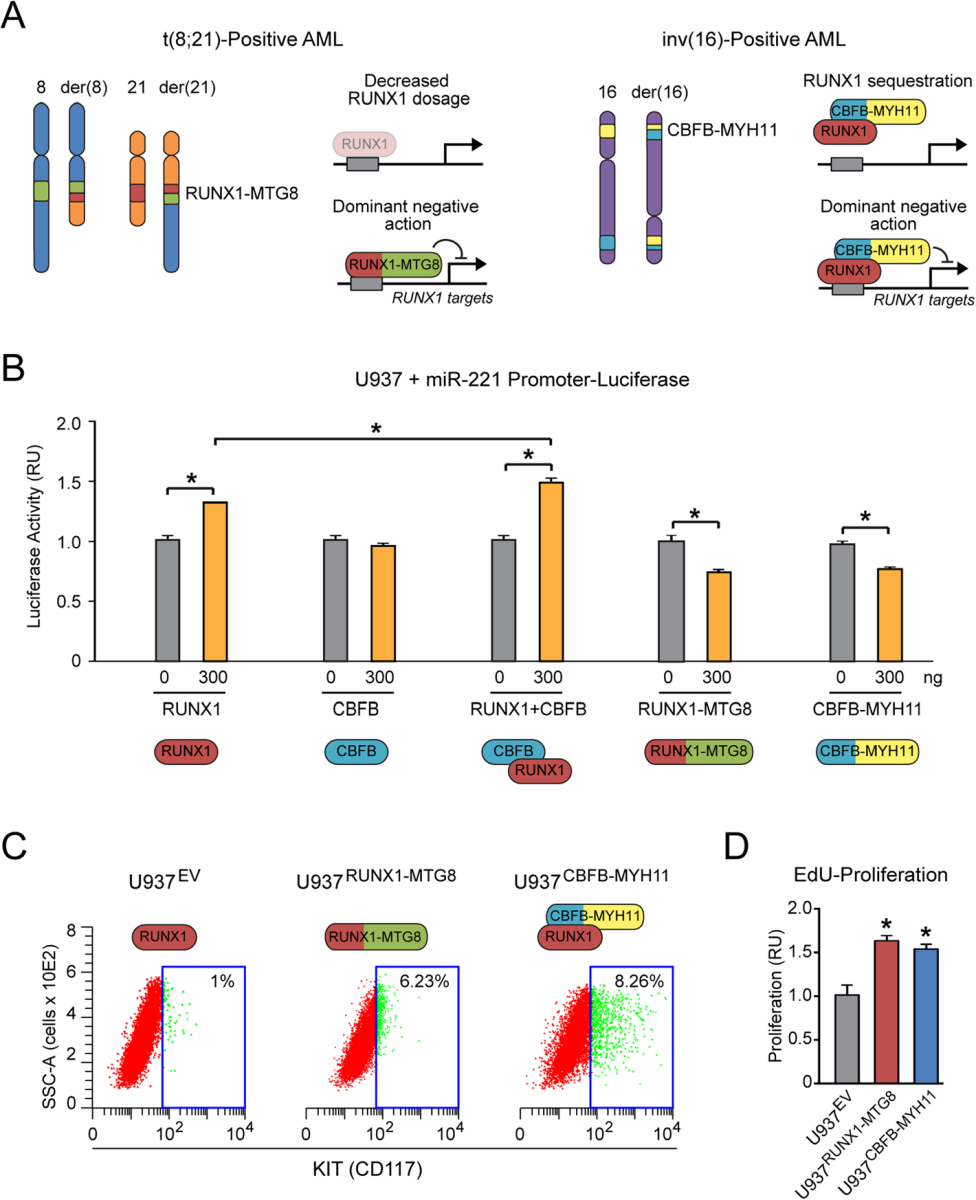
**Both t** **(8;** **21) and inv(16) leukemia fusion proteins affect the same RUNX1-miRNA-KIT axis regulating KIT proliferation activity. (A)** Scheme based on literature showing the mechanisms that affect RUNX1 function in t(8;21) and inv(16) CBF-AML [1,5,8,9]. **(B)** A luciferase reporter driven by the miR-221 promoter is activated by RUNX1, alone or in combination with CBFB, while it is repressed by RUNX1-MTG8 or CBFB-MYH11 in transiently transfected U937 cells. **(C-D)** U937 clones stably expressing either RUNX1-MTG8 (U937^RUNX1-MTG8^) or CBFB-MYH11 (U937^CBFB-MYH11^) display a significant increase (p < 0.05) of KIT-positive cells (assessed by CD117 cytofluorimetric analysis in panel **C**) as well as increased cell proliferation (assessed by EdU incorporation assay in panel **D**). Shown here one representative clone out of 3 clones stably expressing RUNX1-MTG8 or CBFB-MYH11.

Previously, we reported that in t(8;21) and inv(16) CBF-AML samples there is upregulation of KIT (CD117 antigen) concomitant with downregulation of miR-221, a RUNX1-regulated miRNA that targets KIT-3'UTR [17]. Studies from other laboratories showed that the RUNX1-MTG8 fusion protein, derived from the t(8;21) cytogenetic rearrangement, decreases RUNX1 dosage and exerts a dominant negative action over wild type RUNX1 (see scheme in Figure 1A, left, based on [1,5]). The CBFB-MYH11 fusion protein derived from the inv(16) rearrangement would interfere with the wild type RUNX1 function both by depleting the nucleus of RUNX1 through sequestration into the cytoplasm and by exerting a dominant negative action over wild type RUNX1 (Figure 1A, right, based on [8,9]).

Transient transfection of human U937 myeloid cells with a luciferase reporter driven by the miR-221 promoter shows that RUNX1, alone or in combination with CBFB, induces miR-221 transcription, while both RUNX1-MTG8 and CBFB-MYH11 repress miR-221 promoter (Figure 1B). Stable ectopic expression of either RUNX1-MTG8 or CBFB-MYH11 in the U937 cell context increases the proportion of KIT (CD117)-positive cells (Figure 1C) and promotes cell proliferation (Figure 1D), relative to control U937^EV^ cells carrying the cognate empty vector.

Thus, the two CBF-AML fusion proteins, by interfering with wild type RUNX1 transcriptional function, induce miR-221 downregulation concomitant with KIT-induced proliferation.

### Stable expression of miR-17 deregulates the same core RUNX1-miR-221-KIT axis affected by CBF-AML fusion proteins

KIT upregulation was reported in 60-80% of all AML, including CBF-AML and non-CBF-AML [18-21]. Because CBF-AML represents only 15-20% of AML [3,23], we searched for other factors that could explain KIT upregulation in non-CBF-AML. According to *in silico* analysis (TargetScan), the RUNX1-3′UTR contains 22 conserved miRNA binding sites, which are predicted to be targeted by 60 different miRNAs (Additional file 1: Figure S1A, based on Additional file 2: Table S1). Among these, we focused on miR-17-5p (hereafter simply referred to as miR-17), a miRNA that targets RUNX1-3′UTR, plays a key role in RUNX1-mediated control of myeloid differentiation [22], and is often upregulated in leukemia [24].

By examining published miRNA expression datasets from AML patients [24,25], we observed that miR-17 is upregulated in approximately 60% of non-CBF-AML cases, while it is mostly downregulated in CBF-AML cases (Additional file 1: Figure S1B, top). Interestingly, miR-17 upregulation is mostly associated with the FAB M5 subtype (Additional file 1: Figure S1B, bottom), which is frequently characterized by KIT upregulation [18]. Consistent with these observations, we found evidence of concomitant KIT (CD117) and miR-17 upregulation in three out of 10 non-CBF-AML patient samples analyzed in our laboratories (Additional file 1: Figure S1C).

**Figure 2:**
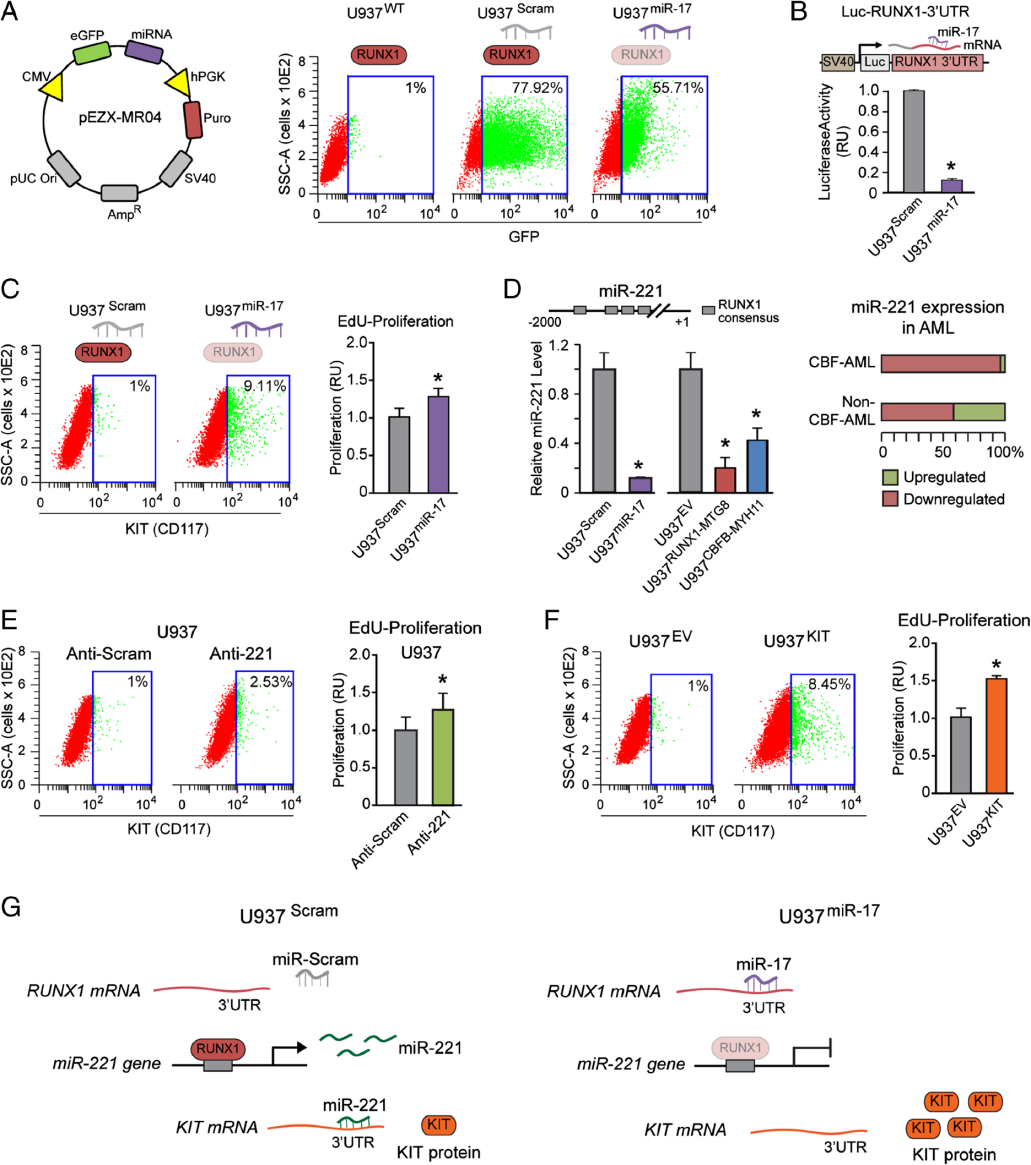
**Stable expression of miR-17 deregulates the same core RUNX1-miR221-KIT axis affected by CBF-AML fusion proteins. (A)** Stable U937 clones ectopically expressing miR-17 (U937^miR-17^) or a control scrambled sequence (U937^Scram^) were developed by transfection with a construct co-expressing GFP and the miRNA precursor of interest (left). Cytofluorimetric analysis shows GFP expression in representative U937^miR-17^ and U937^Scram^ clones (right). **(B)** Stable ectopic expression of miR-17 leads to RUNX1 downregulation, as shown by decreased luciferase activity (bottom) in a representative U937^miR-17^ clone transfected with a reporter plasmid carrying luciferase upstream of RUNX1-3′UTR (top). **(C)** Ectopic expression of miR-17 increases both the number of KIT-positive cells (p < 0.05) (left) and cell proliferation (right). **(D)** Like RUNX1-MTG8 and CBFB-MYH11, miR-17 downregulates miR-221 expression (left). Consistently, both CBF-AML and non-CBF-AML display miR-221 downregulation (right). **(E)** U937 cells transiently transfected with a synthetic anti-miR inhibiting endogenous miR-221 activity display a significant (p <0.01) increase of KIT-positive cells (left) and increased proliferation (right) relative to U937 transiently transfected with a control anti-miR scrambled sequence. **(F)** Stable ectopic expression of wild type KIT in U937 cells (U937^KIT^) (left) is sufficient to promote proliferation (right). **(G)** These results indicate that miR-17, by affecting the RUNX1-miR-221 mechanism, leads to KIT upregulation. Shown here one representative clone out of 3 clones stably expressing RUNX1-MTG8, CBFB-MYH11, or miR-17.

Based on these preliminary observations, we set out to assess the effects of stable ectopic miR-17 expression in U937 cells on both miR-221 and KIT expression. To this end, we stably transfected U937 cells with a plasmid co-expressing a GFP tracking insert adjacent to either the miR-17 precursor or a scrambled control sequence (Figure 2A, left). Next, we selected stable U937^miR-17^ and U937^Scram^ clones positive for GFP expression (Figure 2A, right) and transfected them with a Luc-RUNX1-3′UTR reporter carrying the luciferase sequence upstream of RUNX1-3′UTR (Figure 2B, top). Clones with decreased luciferase expression (a prototypic clone out of three is shown in Figure 2B, bottom) were bona fide miR-17-positive clones. U937^miR-17^ clones showed a higher proportion of KIT (CD117)-positive cells (Figure 2C, left) as well as increased EdU-proliferation (Figure 2C, right) relative to control U937^Scram^ clones.

Apparently, in the U937 cell context miR-17 ectopic expression significantly reduced miR-221 level, thus recapitulating the effect of RUNX1-MTG8 and CBFB-MYH11 (Figure 2D, left). This finding is in agreement with miR-221 downregulation not only in CBF-AML, but also in 50-60% of non-CBF-AML (Figure 2D, right, based on our previous report [17]). Transient transfection of wild type U937 with a synthetic anti-miR-221 targeting endogenous miR-221 induced a significant increase of KIT-positive cells (Figure 2E, left) as well as increased EdU-proliferation (Figure 2E, right). Stable ectopic expression of wild type KIT in the U937 context (U937^KIT^) at a level comparable (5-10%) to the one detected in U937^miR-17^, U937^RUNX1-MTG8^, and U937^CBFB-MYH11^ clones (Figure 2F, left) was *per se* sufficient to increase EdU-proliferation (Figure 2F, right).

Altogether, these findings show that ectopic miR-17 expression deregulates the same RUNX1-miR-221-KIT axis, which is also deregulated by CBF-AML fusion proteins (Figure 2G). Of note, both miR-17 and CBF-AML fusion proteins can affect other RUNX1-regulated miRNAs targeting KIT-3′UTR (see TargetScan analysis in Additional file 3: Table S2). For instance, as shown in Additional file 1: Figure S2, left, miR-193a is significantly downregulated in U937^miR-17^, U937^RUNX1-MTG8^, and U937^CBFB-MYH11^ clones.

### Stable expression of miR-17 downregulates RUNX1-regulated miRNAs of myeloid differentiation

**Figure 3:**
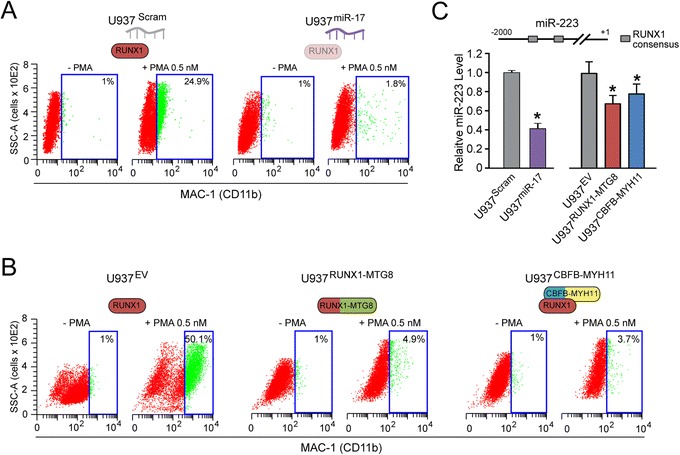
**Stable expression of miR-17 downregulates RUNX1-regulated miRNAs of myeloid differentiation. (A-B)** U937 clones stably expressing miR-17 **(A)**, RUNX1-MTG8, or CBFB-MYH11 **(B)** display block of PMA-induced myeloid differentiation (assessed by CD11b cytofluorimetric analysis) relative to their cognate control clones. **(C)** Consistently, U937^miR-17^, U937^RUNX1-MTG8^, and U937^CBFB-MYH11^ clones also display downregulation of miR-223 (assessed by qRT-PCR).

Treatment with the phorbol ester PMA is a common method to induce U937 cells to rapidly (48-72 h) differentiate into monocytes expressing specific surface myeloid antigens, such as CD11b (Figure 3A and Figure 3B, left). U937^miR-17^ clones (Figure 3A, right), similar to U937^RUNX1-MTG8^ and U937^CBFB-MYH11^ clones (Figure 3B, middle and right), displayed a decrease of CD11b-positive cells in response to PMA, thus indicating myeloid differentiation arrest. Consistently, the U937^miR-17^, U937^RUNX1-MTG8^ and U937^CBFB-MYH11^ clones also displayed significant downregulation of RUNX1-regulated miRNAs involved in myeloid differentiation, such as miR-223 (Figure 3C) and miR-27a (Additional file 1: Figure S2, right) [13,26].

**Figure 4:**
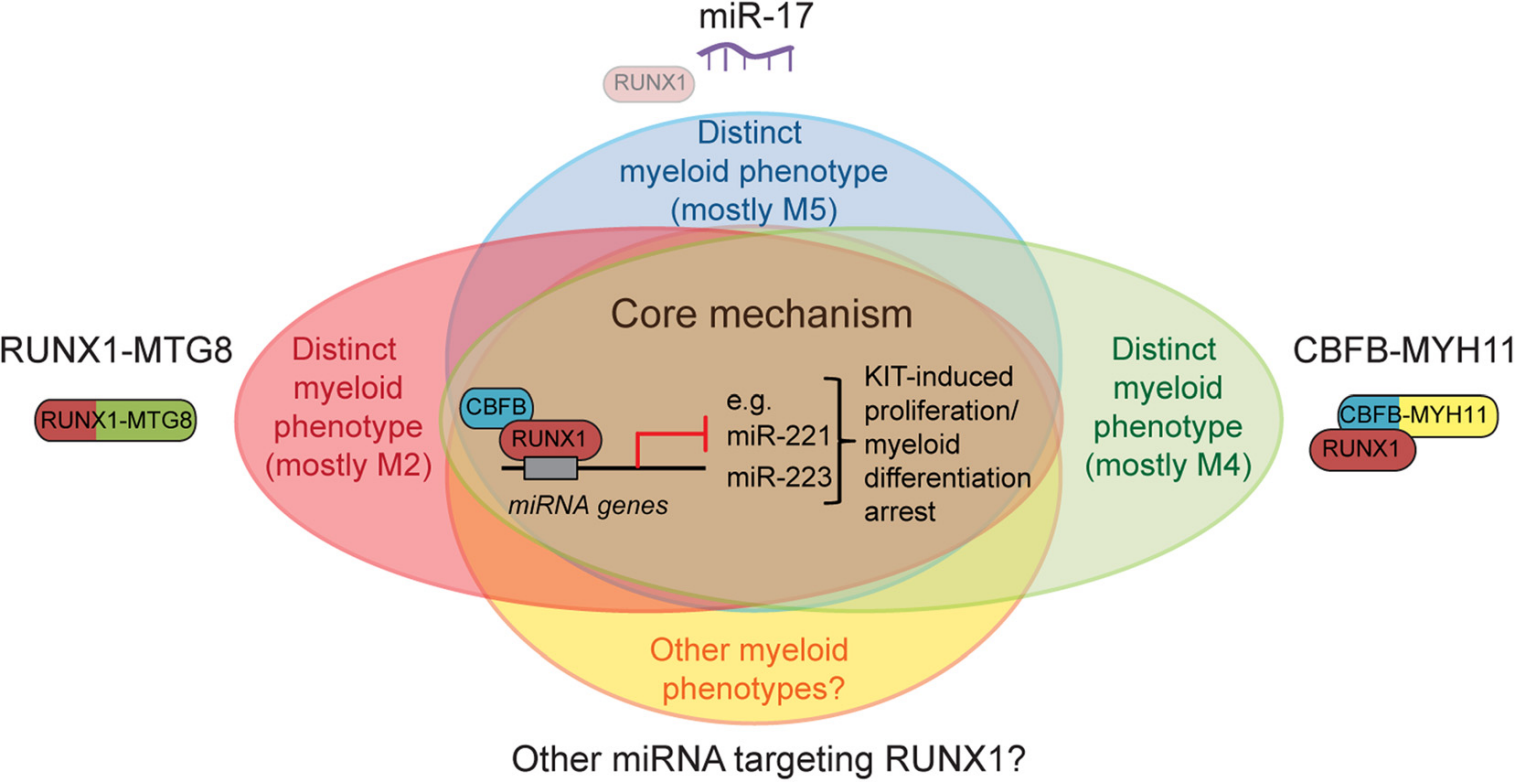
**MiR-17 deregulates a core RUNX1-miRNA mechanism of CBF-AML pathogenesis.** Scheme showing that miR-17 and the RUNX1-MTG8 and CBFB-MYH11 fusion proteins interfere with the same core RUNX1-miRNA mechanism that regulates KIT-mediated proliferation and myeloid differentiation. MiR-17 and the two fusion proteins are expected to produce distinct phenotypes, which may explain their association with different leukemia FAB subtypes. Other miRNAs targeting RUNX1-3′UTR could also affect the RUNX1-miRNA core mechanism.

The overall findings of the first part of this study (schematically summarized in Figure 4) let us conclude that miR-17 deregulates a core RUNX1-miRNA mechanism of CBF-AML pathogenesis, since it recapitulates the same effects of both RUNX1-MTG8 and CBFB-MYH11 fusion proteins. Notably, miR-17 is only one of many miRNAs targeting RUNX1-3′UTR (Additional file 2: Table S1). Some of these miRNAs (e.g. miR-18a, miR-20a, miR-93) are upregulated in a high proportion of non-CBF-AML, and are associated with distinct AML subtypes (Additional file 1: Figure S3). This makes us speculate that these miRNAs, alone or in combination, could also regulate the core RUNX1-miRNA mechanism that governs proliferation and myeloid differentiation.

### Evidence that the RUNX1-miRNA core mechanism is conserved between human and mouse

**Figure 5:**
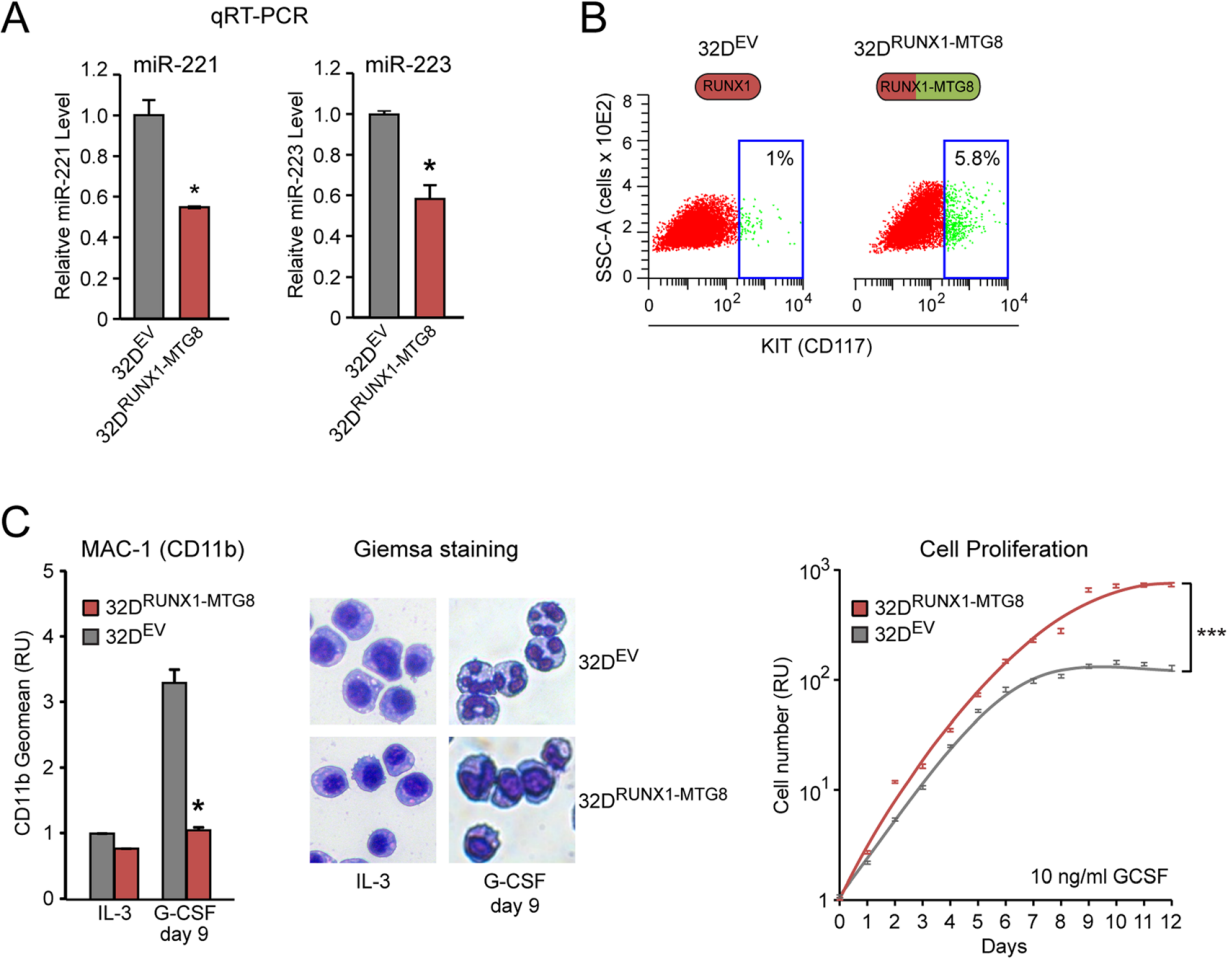
**Evidence that the RUNX1-miRNA core mechanism is conserved between human and mouse. (A)** Stable ectopic expression of RUNX1-MTG8 in 32D myeloid cells (here shown a representative 32D^RUNX1-MTG8^ clone) leads to downregulation of both miR-221 and miR-223 relative to a 32D^EV^ control clone. **(B)** Consistently, CD117 cytofluorimetric analysis shows a significantly (p < 0.05) higher number of KIT-positive cells in 32D^RUNX1-MTG8^ relative to 32D^EV^. **(C)** 32D^RUNX1-MTG8^ display block of granulocytic differentiation in response to G-CSF, as shown by both CD11b cytofluorimetric analysis (left) and Giemsa staining of cytospin preparations (middle), as well as increased proliferation (right) relative to 32D^EV^. Shown here one representative clone for each construct.

Previously we reported that the RUNX1 consensus sequences of the miR-221 and miR-223 promoters, as well as the miR-221 and miR-223 seed sequences, are conserved between human and mouse [17]. Consistently, in mouse myeloid 32D cells, stable expression of RUNX1-MTG8 also leads to downregulation of miR-221 and miR-223 (Figure 5A). Concomitantly, 32D^RUNX1-MTG8^ cells also display an increased proportion of KIT-positive cells (Figure 5B), and are no longer able to undergo myeloid differentiation in response to granulocyte colony stimulating factor (G-CSF). Indeed, in the presence of G-CSF (10 ng/ml), 32D^RUNX1-MTG8^ cells do not acquire changes typical of granulocytes, such as increased CD11b expression (Figure 5C, left) and nuclear segmentation (Figure 5C, middle). Moreover, in contrast to control 32D^EV^ cells, 32D^RUNX1-MTG8^ cells continue to proliferate even after day 7 (Figure 5C, right).

These findings let us hypothesize that the RUNX1-miRNA-KIT mechanism of proliferation can have an influence on the myeloid differentiation process. Thus, in the second part of this study, we took advantage of the 32D cell model to test whether KIT-mediated proliferation can *per se* affect myeloid differentiation.

### The extent of KIT expression *per se* delays myeloid differentiation in cells with functional RUNX1

**Figure 6:**
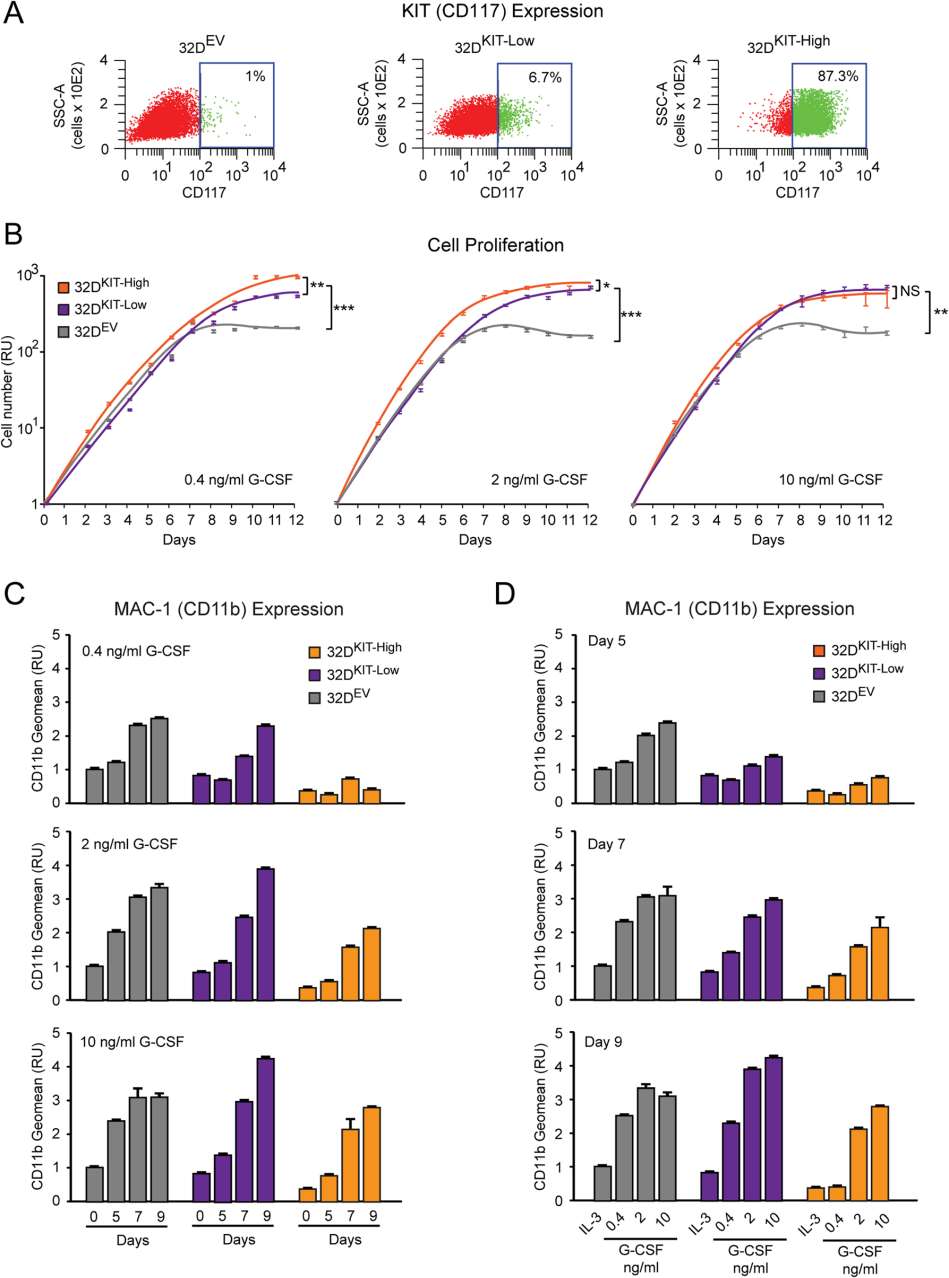
**The extent of KIT expression** ***per se*** **delays myeloid differentiation in cells with functional RUNX1. (A)** CD117 cytofluorimetric analysis showing ectopic KIT expression in 32D^KIT-Low^ and 32D^KIT-High^ stable clones. **(B)** Growth curves showing increased proliferation of 32D^KIT-Low^ and 32D^KIT-High^ clones relative to the 32D^EV^ control clone in response to different concentrations of G-CSF. **(C-D)** CD11b cytofluorimetric analysis shows that increasing KIT expression delays G-CSF-induced differentiation in a G-CSF dose-dependent **(C)** and time-dependent manner **(D)**. See text and Additional file 4: Table S3 for statistical significance.

To mechanistically assess whether increasing KIT expression *per se* can affect G-CSF-induced differentiation, we developed 32D clones expressing exogenous mouse wild type KIT (assessed as CD117 antigen) at low (5-10%) or high (>80%) level relative to control 32D^EV^ cells (Figure 6A). Specifically, we used a 32D^KIT-Low^ clone expressing KIT at a level comparable to the 32D^RUNX1-MTG8^ clone (shown in Figure 5B), and a 32D^KIT-High^ clone expressing KIT at a level comparable to a t(8;21)-positive leukemia cell line, the Kasumi cell line (data not shown).

32D^EV^, 32D^KIT-Low^, and 32D^KIT-High^ cells treated with increasing concentrations of G-CSF (0.4, 2, and 10 ng/ml) were analyzed for both cell proliferation and induction of CD11b myeloid antigen over a 12 day period. Control 32D^EV^ cells proliferated exponentially up to day 7 before plateauing and dying (Figure 6B, gray line). Concomitantly, CD11b expression increased in a time-dependent and G-CSF dose-dependent manner. By day 9, almost all 32D^EV^ cells reached full differentiation (Figure 6C and 6D, gray bars). Upon treatment with G-CSF, both 32D^KIT-Low^, and 32D^KIT-High^ cells displayed significantly increased proliferation relative to control 32D^EV^ cells, particularly at the lower G-CSF concentrations (Figure 6B and Additional file 4: Table S3).

When we analyzed CD11b expression at key time points of G-CSF-induced differentiation (5, 7, and 9 days), the most significant differences were detected between 32D^KIT-High^ and 32D^EV^ clones. First, by taking into consideration individual G-CSF concentrations (Figure 6C and Additional file 4: Table S3), it was apparent that 32D^KIT-High^ cells failed to differentiate at the lowest (0.4 ng/ml) G-CSF concentration across all time points (p = 6.7^−7^), while they were able to differentiate to a level similar to the 32D^EV^ clone by day 9 at the highest (10 ng/ml) G-CSF concentration (p = 0.91). Second, by taking into consideration individual time points (Figure 6D and Additional file 4: Table S3), it was apparent that the 32D^KIT-High^ cells had significantly lower CD11b on day 5 (p = 6^−5^) and day 7 (p = 0.02) across all G-CSF concentrations, but they reached a level of differentiation similar to the 32D^EV^ clone (p = 0.12) on day 9, at the higher G-CSF concentrations. Finally, when we took into consideration the level of exogenous KIT expression, 32D^KIT-Low^ cells displayed a G-CSF differentiation response which was intermediate between 32D^EV^ and 32D^KIT-High^ cells (Figure 6C and 6D).

These findings indicate that the extent of KIT-induced proliferation *per se* plays a critical role in delaying G-CSF-induced myeloid differentiation.

### Inhibition of KIT activity counteracts the delay effect on myeloid differentiation due to KIT overexpression

**Figure 7:**
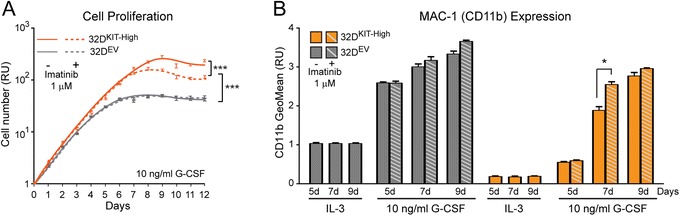
**Inhibition of KIT activity counteracts the delay effect on myeloid differentiation due to KIT overexpression. (A)** Imatinib (1 μM) significantly decreases the proliferation of 32D^KIT-High^ cells during G-CSF-induced differentiation, while it does not affect control 32D^EV^ cells. **(B)** CD11b cytofluorimetric analysis at different time points during G-CSF-induced differentiation showing that the KIT-induced delay of myeloid differentiation in 32D^KIT-High^ cells is counteracted by imatinib. See Additional file 4: Table S3 for statistical significance.

We assessed whether inhibition of KIT proliferation activity could counteract G-CSF-induced myeloid differentiation by treating 32D^KIT-High^ cells with imatinib (1 μM), a receptor tyrosine kinase inhibitor targeting KIT [27]. Imatinib significantly reduced the proliferation of 32D^KIT-High^ cells relative to cells grown only in the presence of G-CSF (10 ng/ml), but did not significantly affect the proliferation of 32D^EV^ control cells (Figure 7A and Additional file 4: Table S3). This indicates that the anti-proliferative effect of imatinib was likely due to specific inhibition of KIT activity. By measuring CD11b at various time points (day 5, 7, and 9) during G-CSF treatment, we found that imatinib did not significantly change CD11b level in 32D^EV^ control cells, but increased the CD11b level of the 32D^KIT-High^ cells to the level detected in the 32D^EV^ cells by day 7 (Figure 7B). Thus, imatinib was able to counteract the KIT-induced delay of myeloid differentiation.

**Figure 8:**
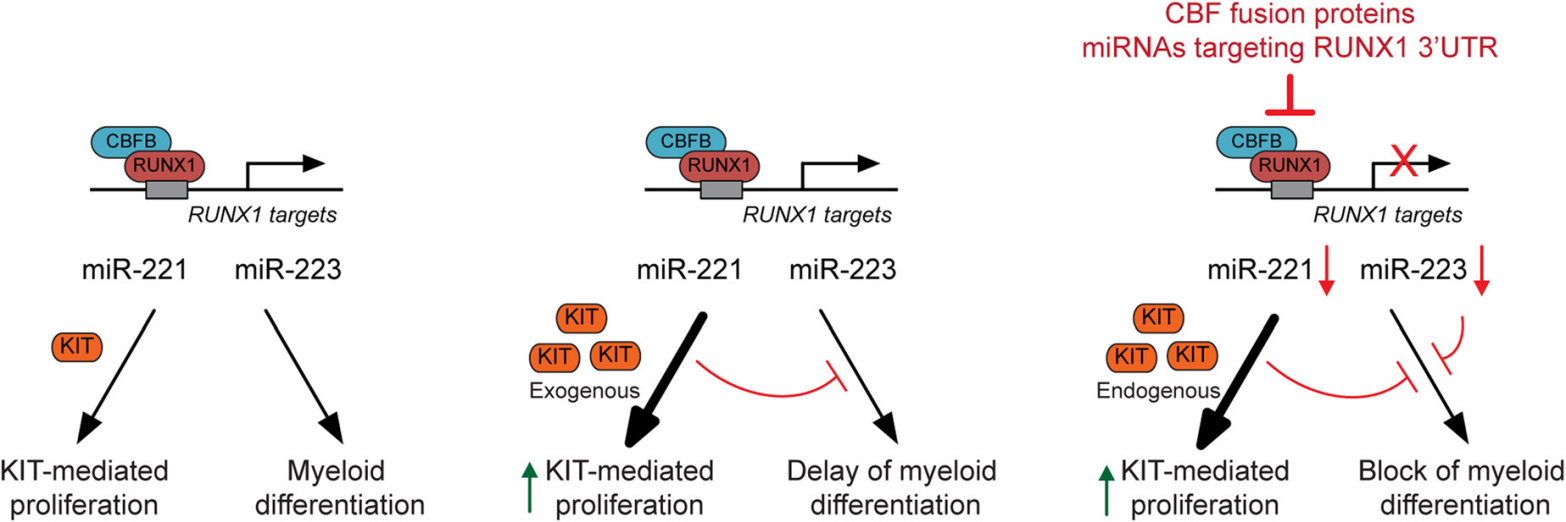
**RUNX1 regulates myeloid differentiation by modulating the extent of KIT-induced proliferation.** In the presence of a functional RUNX1-miRNA mechanism, cells respond to myeloid differentiation stimuli, such as G-CSF (left). Increasing KIT-mediated proliferation, even in the absence of factors interfering with RUNX1, is sufficient to delay myeloid differentiation (middle). In the presence of factors that impair the RUNX1-miRNA mechanism (e.g. CBF fusion proteins or miRNAs targeting RUNX1-3′UTR), the effect of KIT-mediated proliferation reinforces the effects of deregulation of miRNAs controlled by RUNX1 and involved in differentiation (e.g. miR-223), thus resulting in complete block of myeloid differentiation (right).

Based on the overall findings in the 32D mouse model, we conclude that the proliferation effect of increased KIT can *per se* delay myeloid differentiation. In the presence of factors that impair RUNX1 function/level, this delaying effect likely reinforces the effects of direct deregulation of RUNX1-regulated target genes (e.g. miR-223) involved in myeloid differentiation (Figure 8).

## Discussion

In the first part of this study, we show that ectopic expression of miR-17, which controls RUNX1 level by targeting RUNX1-3′UTR [22], in human U937 cells leads to deregulation of a core RUNX1-regulated miRNA mechanism that is similarly affected by the t(8;21) RUNX1-MTG8 and inv(16) CBFB-MYH11 fusion proteins. Apparently, KIT receptor upregulation in CBF-AML and non-CBF-AML can be induced not only by genetic factors (CBF-AML fusion proteins), but also epigenetic factors (miRNAs targeting RUNX1-3′UTR) that interfere with wild type RUNX1 function.

Notably, acute myeloid leukemia with t(8;21), inv(16), or upregulation of miR-17 fall into FAB subtypes with distinct phenotypic features. It is plausible that these phenotypic differences are due to distinct mechanisms of the different factors that negatively affect RUNX1. For instance, RUNX1-MTG8 and CBFB-MYH11, which are mostly associated with the M2 and M4Eo FAB subtypes, respectively, exert dominant negative effects over wild type RUNX1 through different mechanisms [1,5,8,9]. Moreover, RUNX1-MTG8 (but not CBFB-MYH11) can bind other MTG proteins [28]. In the case of miR-17 overexpression, which is mainly associated with the M5 FAB subtype, the phenotype could be due to deregulation of other miR-17 targets, besides RUNX1.

In addition to CBF-AML fusion proteins and miR-17, other factors could deregulate RUNX1 function or level. For instance, *in silico* analysis of published miRNA expression datasets allowed us to highlight several miRNAs that could potentially target RUNX1-3′UTR and that, like miR-17, are upregulated in non-CBF-AML. Further, deregulation of canonical RUNX1 transcriptional regulators (e.g. GATA-2 and regulatory Ets proteins) and even long non-coding RNAs affecting RUNX1 chromatin state, such as the recently discovered RUNXOR [29], may ultimately affect the core RUNX1-miRNA mechanism that regulates myeloid cell proliferation and differentiation.

Proliferation and differentiation are intimately linked processes, and deregulation of one can impact the other [30,31]. In the second part of this study, by taking advantage of the conservation of the core RUNX1-miRNA-KIT axis between human and mouse myeloid cells, we found evidence that KIT-induced proliferation can *per se* influence G-CSF-induced granulocytic differentiation of 32D myeloid cells. Specifically, increasing KIT level negatively affects 32D myeloid differentiation both in a time- and G-CSF dose-dependent manner. Therefore, the extent of KIT pro-proliferative signaling can oppose differentiation signalings, such as the G-CSF signaling. Indeed, modulation of KIT-mediated proliferation with imatinib, an inhibitor of KIT receptor activity, favors G-CSF-induced 32D differentiation. In contrast, stable expression of RUNX1-MTG8, which irreversibly affects the RUNX1-miRNA-KIT mechanism, promotes the proliferation of myeloid cells that are unresponsive to G-CSF. In the latter case, the delay of GCSF-induced differentiation due to KIT proliferation is reinforced by the repression of RUNX1-regulated coding and non-coding (e.g. miR-223) differentiation functions (Figure 8).

## Conclusions

In conclusion, on the one hand, miRNA-mediated deregulation of RUNX1 function mimics the effects of CBF-AML fusion proteins by affecting a core RUNX1-miRNA mechanism of KIT-induced proliferation of undifferentiated myeloid cells. On the other hand, the extent of KIT-induced proliferation itself can modulate the process of differentiation of myeloid cells with normal RUNX1 function.

## Methods

### AML patient samples

Patient samples were obtained in compliance with the Helsinki declaration as per Institutional Review Board guidelines of the Niguarda Hospital, Milan, Italy. Leukemic mononuclear cells (MNCs), isolated from the bone marrow of 10 patients with de novo non-CBF-AML according to the French-British-American (FAB) classification, did not show cytogenetic evidence of CBF rearrangements. Samples were analyzed for KIT (CD117) expression by flow cytometry and for miR-17 expression by qRT-PCR. DNA sequencing of RUNX1 exons excluded the presence of mutations in those samples showing upregulation of both KIT and miR-17.

### *In silico* analyses

The 3′UTRs of human RUNX1 mRNA (NM_001001890) and human KIT mRNA (NM_001093772) were analyzed by using TargetScan Version 6.2: June 2012 (http://www.targetscan.org). The predicted miRNA-targeting sites conserved among vertebrates were chosen to generate a list of putative miRNAs targeting the RUNX1-3′UTR (Additional file 2: Table S1) or KIT-3′UTR (Additional file 3: Table S2). The expression of miR-221 in AML patient samples (12 non-CBF-AML and 27 CBF-AML) was evaluated from our previous miRNA expression data [17]. The expression of miR-17, miR-18a, miR-20a, miR-93, and miR-181 in AML patient samples was evaluated from published gene expression datasets [24,25]. Specifically, 52 non-CBF-AML and 31 CBF-AML were analyzed for miR-17, 31 non-CBF-AML and 18 CBF-AML were analyzed for miR-18a, 53 non-CBF-AML and 34 CBF-AML were analyzed for miR-20a, 34 non-CBF-AML and 18 CBF-AML were analyzed for miR-93 and miR-181. The miRNAs were classified as upregulated, downregulated, or unchanged relative to the centered mean and plotted by AML subtype.

### Plasmid cloning

Human RUNX1-MTG8 cDNA was PCR-amplified from pcDNA3.1-RUNX1-MTG8 [28], with primers introducing Xho I and Not I restriction sites (forward: 5′ – AGA TCT CGA GAT GCG TAT CCC CGT AGA TGC – 3′; reverse: 5′ – GAC AAG CGG CCG CCT AGC GAG GGG TTG TCT CTA – 3′), and inserted into compatible restriction sites of the pLNCX2 plasmid. Human CBFB-MYH11 cDNA was subcloned from the pGEM-CMVa-CBFB-MYH11 plasmid (kindly provided by Dr. Paul Liu, NIH, Bethesda, MD) by restriction digestion using Hind III and Not I restriction enzymes (New England BioLabs, Ipswich, MA) and inserted into compatible restriction sites of pLNCX2. The mouse KIT cDNA was subcloned from pCMV-Sport6-c-Kit (GE Dharmacon, Lafayette, CO) into compatible restriction sites of pLNCX2. The human KIT cDNA was PCR-amplified from pDNR-KIT (Dana-Farber/Harvard Cancer Center DNA Resource Core) with primers introducing Xho I and Not I restriction sites (forward: 5′ - AGA TCT CGA GAC CAT GAG AGG CGC TCG CGG CGC CT – 3′; reverse: 5′ – ACC TGC GGC CGC TCA GAC ATC GTC GTG CAC AAG C – 3′) and inserted into compatible restriction sites of pLNCX2. All plasmids were sequenced to confirm the absence of mutations.

### Stable cell clones and culture conditions

Human leukemic monocyte lymphoma U937 cells and derived clones were cultured in RPMI 1640 supplemented with 10% heat-inactivated fetal bovine serum (Life Technologies, Carlsbad, CA). Mouse myeloid 32D/WT1 cells, ectopically expressing human granulocyte colony-stimulating factor receptor (G-CSFR) [32], and derived clones were cultured in RPMI 1640 medium supplemented with 10% heat-inactivated fetal bovine serum (Life Technologies) supplemented with 10 ng/ml of mouse IL-3 (PeproTech, Rocky Hill, NJ). All cultures were maintained at a concentration between 0.2 × 10^6^-1.0 × 10^6^ cells/ml.

U937 and 32D stable clones were obtained by stable transfection with pLNCX2-RUNX1-MTG8, pLNCX2-CBFB-MYH11, or pLNCX2-KIT using Lipofectamine LTX with Plus reagent (Life Technologies), according to the manufacturer’s instructions. In parallel, cells were also transfected with the cognate pLNCX2 empty vector to obtain control clones. After 48 hours of transfection, cells were seeded in limiting dilutions in 96-well plates and selected with 1 mg/ml of G418. Single clones were expanded and screened for the presence of the transfected construct by PCR. Expression and function of RUNX1-MTG8 or CBFB-MYH11 in positive clones was confirmed by RT-PCR with specific primers [33], and the effect on transcription of known RUNX1-target genes (e.g. MPO, CSF1R). Expression of exogenous KIT was assessed by cytofluorimetric analysis as described below.

To develop U937 clones stably expressing ectopic miR-17 or cognate control clones, cells were transfected with pEZX-MR04 plasmid (GeneCopoeia, Rockville, MD) containing either the miR-17 precursor or a scrambled sequence, respectively. Single clones were selected with 1 μg/ml of puromycin and screened for expression of GFP, which is constitutively expressed from the pEZX-MR04, by cytofluorimetric analysis. GFP-positive clones showing significant downregulation of luciferase-RUNX1-3′UTR were selected for further analysis. Only prototypic clones are shown in the figures of this manuscript.

### Flow cytometry analysis

AML patient samples were analyzed for KIT (CD117) expression as previously described [17]. For cytofluorimetric analysis of U937 and 32D clones, 1 × 10^6^ cells were resuspended in blocking buffer (PBS + 0.5% BSA) in a total volume of 100 μl. After adding fluorescently labeled antibodies against human KIT (BV-CD117, BD Biosciences, San Jose, CA), mouse KIT (PE-CD117, Miltenyi Biotec, San Diego, CA), or mouse/human CD11b (FITC-CD11b or PE-CD11b, Miltenyi Biotec), cells were incubated for 20 minutes at 4°C in the dark. Unstained cells were used as a negative control. After incubation, cells were washed, resuspended in 500 μl of blocking buffer and analyzed by flow cytometry on a LSR Fortessa cytometer (BD Biosciences). Data were analyzed using WinList software. Statistical significance was calculated by using the Student’s *t*-test.

### EdU cell proliferation assay

Cell proliferation was assessed by EdU incorporation, using the Click-iT EdU imaging kit (Life Technologies) as per manufacturer’s instructions. Briefly, 0.2 × 10^6^ cells/ml were seeded in fresh growth medium, let grow for 48 h, incubated with 10 μM EdU for 20 minutes, and transferred to glass microscope slides using a cytospin. The slides were fixed and permeabilized with 100% Methanol, incubated for 30 minutes in PBS containing 3% BSA, reacted with the Click-iT reaction cocktail (containing azide-conjugated Alexa Fluor 594) for 30 minutes, counterstained with DAPI, and mounted with Vectashield (Vector Laboratories, Burlingame, CA). Slides were examined by fluorescence microscopy to count the EdU-positive and DAPI-positive nuclei of at least 10 random fields. Proliferation was evaluated by calculating the ratio between EdU-positive and DAPI-positive cells. Statistical significance was calculated by using the Student’s *t*-test.

### Quantitative real-time RT-PCR (qRT-PCR)

Total RNA was isolated from cells with the single-step method [34] using Trizol (Life Technologies). 50 ng of total RNA was retrotranscribed into cDNA by using the TaqMan® miRNA Reverse Transcription kit and TaqMan® assay RT primers specific for each of the miRNA of interest, according to the manufacturer’s instructions (Life Technologies). A TaqMan® assay RT primer for RNU-44, a human small nucleolar RNA, was used as an internal loading control for human cells, and a TaqMan® assay RT primer for sno-202, a mouse small nucleolar RNA, was used as an internal loading control for mouse cells. The levels of miRNA cDNAs were quantitated by real-time PCR analysis using specific TaqMan® assay real-time primers (Life Technologies) for each miRNA of interest, as well as for the small nucleolar RNA internal loading controls. Real-time PCR was performed on an iCycler (BioRad, Hercules, CA) using the iQ Supermix (BioRad) and quantitated from triplicate readings using the delta-delta Ct method. Statistical significance was calculated by the Student’s *t*-test.

### Luciferase reporter assays

For miR-221-promoter luciferase assay, U937 cells grown in a 24-well plate were transfected by using Lipofectamine LTX (Life Technologies) with 10 ng of pRL-TK (Promega, Madison, WI), 300 ng of (-1600) MIR-222/221-Luc (kindly provided by Carlo Croce, Ohio State University), in combination with 300 ng of either pCMV5-RUNX1B (Addgene, Cambridge, MA), pcDNA3.1-AML1-MTG8-V5 [28], pGEM-CMV-CBFB, or pGEM-CMV-CBFB-MYH11 (both kindly provided by Paul Liu, NIH, Bethesda, MD). For RUNX1-3′UTR luciferase assay, cells seeded into 24-well plates were co-transfected with 10 ng of the pRL-TK and either 500 ng of pGL4.13-RUNX1-3′UTR plasmid, containing the RUNX1-3′UTR downstream of the firefly luciferase coding sequence (kindly provided by Yoram Groner, Weizmann Institute, Israel), or 500 ng of the control pGL4.13 plasmid. The concentration of transfected DNA was kept constant by adding an appropriate amount of empty pcDNA3.1. After 24 hours, cells were lysed in passive lysis buffer and measured for luciferase activity using the Dual Glow Luciferase Assay System (Promega) and a Veritas Luminometer (Tuner Biosystems, Sunnyvale, CA) as per manufacturer′s instructions. Statistical significance was calculated by using the Student’s *t*-test.

### Differentiation assays

To induce U937 monocytic differentiation, cells were treated with 0.5 nM PMA (Sigma, St Louis, MO), for 48 hours. Differentiation was assessed by cytofluorimetric analysis of the CD11b myeloid marker. Granulocytic differentiation of 32D cells was induced by replacing IL-3 with human G-CSF (Amgen, Thousand Oaks, CA) at the indicated concentrations. Cells were counted daily using a Beckman Coulter cell counter to obtain a growth curve and diluted daily to 0.2 × 10^6^ cells/ml. Differentiation was either microscopically evaluated on Giemsa-stained cytospin preparations, or assessed by CD11b cytofluorimetric analysis. Treatments with imatinib (Cayman Chemical, Ann Arbor, MI) were performed at 1 μM concentration in the G-CSF containing medium, based on [35]. Differences in proliferation or CD11b levels between clones were analyzed by performing ANOVA statistical analysis. For CD11b, ANOVA analysis was performed between dose and clone conditional on time, and time and clone conditional on dose.

## Additional files

Additional file 1: Figure S1. Evidence of concomitant upregulation of KIT and miR-17 in non-CBF-AML (A) Scheme showing the putative miRNA-target sites in the RUNX1-3′UTR, as predicted by TargetScan analysis. (B) Analysis of published miRNA data sets [24,25] from AML patient samples showing that miR-17 is upregulated in non-CBF-AML relative to CBF-AML (top); the upregulation is mostly observed in FAB M5 AML (bottom). (C) Analysis of leukemic mononuclear cells isolated from the bone marrow of non-CBF-AML patients showing concomitant upregulation of KIT (measured by flow cytometry) and miR-17 (measured by qRT-PCR) in three out of 10 cases. **Figure S2.** Additional miRNAs regulated by RUNX1 and deregulated by ectopic expression of CBF-AML fusion proteins or miR-17. Stable ectopic expression of RUNX1-MTG8, CBFB-MYH11, or miR-17 in U937 cells (a representative U937 clone is shown for each construct) leads to downregulation of miR-193a, a RUNX1-regulated miRNA targeting KIT (left), and miR-27a, a RUNX1-regulated miRNA involved in myeloid differentiation (right). **Figure S3.** Additional miRNAs regulated by RUNX1 showing upregulation in non-CBF-AML (A) In addition to miR-17, Targetscan analysis identifies several other miRNAs targeting RUNX1-3′UTR. (B) Analysis of published miRNA data sets [24,25] from AML patient samples shows that a few of these miRNAs are upregulated in non-CBF-AML and are associated with distinct AML FAB subtypes. Additional file 2: Table S1. List of miRNAs predicted to target RUNX1-3′UTR by Targetscan analysis. Additional file 3: Table S2. List of miRNAs predicted to target KIT 3′UTR by Targetscan analysis. Additional file 4: Table S3. Statistical analysis of 32D growth curves and CD11b expression.
