# Supplementary material for: miR-17 deregulates a core RUNX1-miRNA mechanism of CBF acute myeloid leukemia
Source: Mol Cancer. 2015 Jan 23;14:7. doi: 10.1186/s12943-014-0283-z (PMC4351976; doi:10.1186/s12943-014-0283-z)
Supplement: Additional file 1: Figure S1. — Evidence of concomitant upregulation of KIT and miR-17 in non-CBF-AML (A) Scheme showing the putative miRNA-target sites in the RUNX1-3′UTR, as predicted by TargetScan analysis. (B) Analysis of published miRNA data sets [24,25] from AML patient samples showing that miR-17 is upregulated in non-CBF-AML relative to CBF-AML (top); the upregulation is mostly observed in FAB M5 AML (bottom). (C) Analysis of leukemic mononuclear cells isolated from the bone marrow of non-CBF-AML patients showing concomitant upregulation of KIT (measured by flow cytometry) and miR-17 (measured by qRT-PCR) in three out of 10 cases. Figure S2. Additional miRNAs regulated by RUNX1 and deregulated by ectopic expression of CBF-AML fusion proteins or miR-17. Stable ectopic expression of RUNX1-MTG8, CBFB-MYH11, or miR-17 in U937 cells (a representative U937 clone is shown for each construct) leads to downregulation of miR-193a, a RUNX1-regulated miRNA targeting KIT (left), and miR-27a, a RUNX1-regulated miRNA involved in myeloid differentiation (right). Figure S3. Additional miRNAs regulated by RUNX1 showing upregulation in non-CBF-AML (A) In addition to miR-17, Targetscan analysis identifies several other miRNAs targeting RUNX1-3′UTR. (B) Analysis of published miRNA data sets [24,25] from AML patient samples shows that a few of these miRNAs are upregulated in non-CBF-AML and are associated with distinct AML FAB subtypes. [file 12943_2014_283_MOESM1_ESM.pdf]

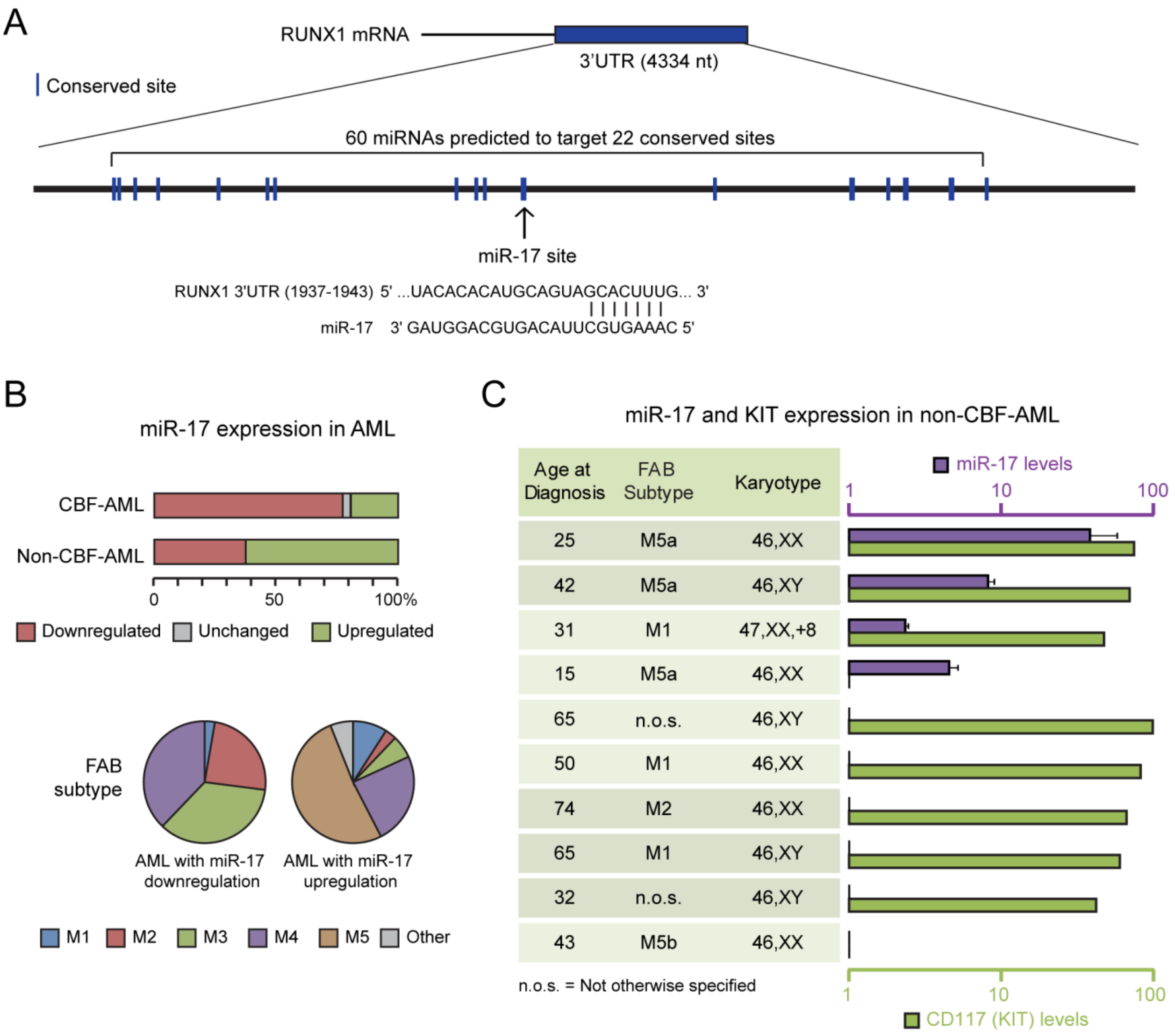

Figure S1. Evidence of concomitant upregulation of KIT and miR-17 in non-CBF-AML
(A) Scheme showing the putative miRNA-target sites in the RUNX1-3'UTR, as predicted by TargetScan analysis. (B) Analysis of published miRNA data sets [24, 25] from AML patient samples showing that miR-17 is upregulated in non-CBF-AML relative to CBF-AML (top); the upregulation is mostly observed in FAB M5 AML (bottom). (C) Analysis of leukemic mononuclear cells isolated from the bone marrow of non-CBF-AML patients showing concomitant upregulation of KIT (measured by flow cytometry) and miR-17 (measured by qRT-PCR) in three out of 10 cases.

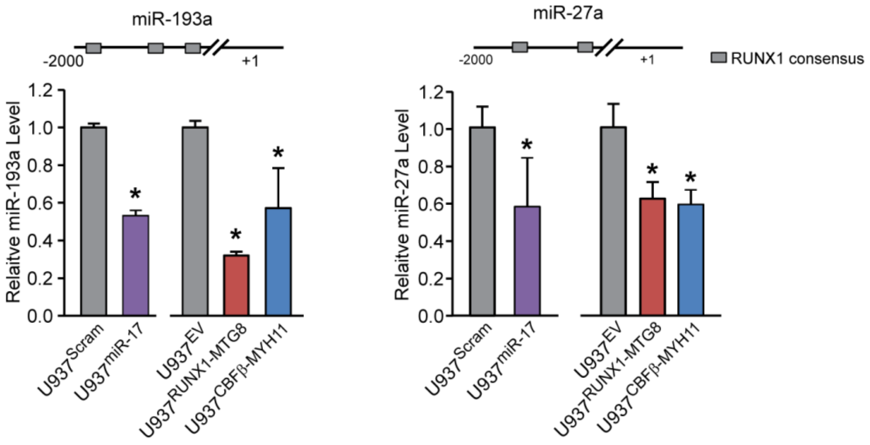

Figure S2. Additional miRNAs regulated by RUNX1 and deregulated by ectopic expression of CBF-AML fusion proteins or miR-17

Stable ectopic expression of RUNX1-MTG8, CBFB-MYH11, or miR-17 in U937 cells (a representative U937 clone is shown for each construct) leads to downregulation of miR-193a, a RUNX1-regulated miRNA targeting KIT (left), and miR-27a, a RUNX1-regulated miRNA involved in myeloid differentiation (right).

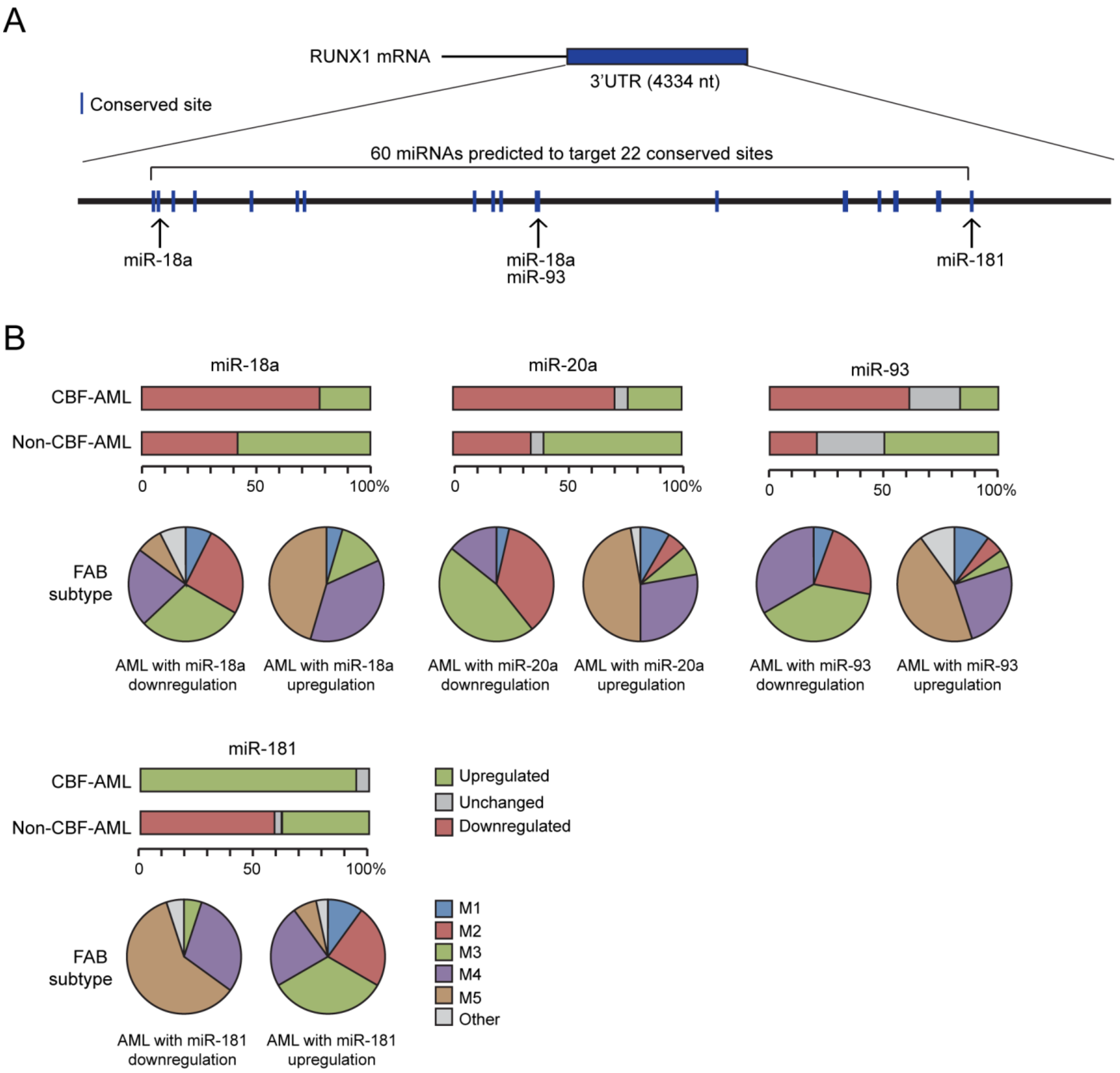

Figure S3. Additional miRNAs regulated by RUNX1 showing upregulation in non-CBF-AML

(A) In addition to miR-17, Targetscan analysis identifies several other miRNAs targeting RUNX1-3'UTR. (B) Analysis of published miRNA data sets [24, 25] from AML patient samples shows that a few of these miRNAs are upregulated in non-CBF-AML and are associated with distinct AML FAB subtypes.
