# Supplementary material for: miR-17 deregulates a core RUNX1-miRNA mechanism of CBF acute myeloid leukemia
Source: Mol Cancer. 2015 Jan 23;14:7. doi: 10.1186/s12943-014-0283-z (PMC4351976; doi:10.1186/s12943-014-0283-z)
Supplement: Additional file 2: Table S1. — List of miRNAs predicted to target RUNX1-3′UTR by Targetscan analysis. [file 12943_2014_283_MOESM2_ESM.pdf]

Supplementary Table S1. MicroRNAs predicted to target RUNX1 3' UTR (NM_001001890 - 4334 nt) by TargetScan analysis

<table>
<tr><th>MicroRNA</th><th>Position in RUNX1 3'UTR</th><th>predicted pairing of target region (top) and miRNA (bottom)</th><th>seed match</th><th>context + score</th><th>context + score percentile</th><th>PCT</th></tr>
<tr><td>hsa-miR-27a</td><td>321-328</td><td>5' ...GAUUCCCGGAGGGAAACUGUGAA...
|||||||
3' CGCCUUGAAUCGGUGACACUU</td><td>8mer</td><td>-0.24</td><td>91</td><td>0.9</td></tr>
<tr><td></td><td>348-354</td><td>5' ...UCUGAUUUAGCAAUGCUGUGAAU...
||||| ||||||
3' CGCCUUGAAUCGGU--GACACUU</td><td>7mer-1A</td><td>-0.13</td><td>73</td><td>0.7</td></tr>
<tr><td>hsa-miR-27b</td><td>321-328</td><td>5' ...GAUUCCCGGAGGGAAACUGUGAA...
|||||||
3' CGUCUUGAAUCGGUGACACUU</td><td>8mer</td><td>-0.23</td><td>90</td><td>0.9</td></tr>
<tr><td></td><td>348-354</td><td>5' ...UCUGAUUUAGCAAUGCUGUGAAU...
||||| ||||||
3' CGUCUUGAAUCGGU--GACACUU</td><td>7mer-1A</td><td>-0.13</td><td>73</td><td>0.7</td></tr>
<tr><td>hsa-miR-128</td><td>321-327</td><td>5' ...GAUUCCCGGAGGGAAACUGUGAA...
||||||
3' UUUCUCUGGCCAAGUGACACU</td><td>7mer-1A</td><td>-0.07</td><td>50</td><td>0.79</td></tr>
<tr><td>hsa-miR-9</td><td>408-414</td><td>5' ...ACCAAGUUGUUUAUUCCAAAGAG...
||||||
3' AGUAUGUCGAUCUAUUGGUUUCU</td><td>7mer-1A</td><td>-0.06</td><td>54</td><td>0.61</td></tr>
<tr><td>hsa-miR-4735-3p</td><td>498-505</td><td>5' ...AUUUUUAAUUUUUCCGCACCUUA...
|||||| |||||||
3' UACAGAUUAAACU--CGUGGAAA</td><td>8mer</td><td>-0.39</td><td>97</td><td>0.78</td></tr>
<tr><td>hsa-miR-18a</td><td>498-505</td><td>5' ...AUUUUUAAUUUUUCCGCACCUUA...
|||||||
3' GAUAGACGUGAUCUACGUGGAAU</td><td>8mer</td><td>-0.31</td><td>93</td><td>0.78</td></tr>
<tr><td>hsa-miR-18b</td><td>498-505</td><td>5' ...AUUUUUAAUUUUUCCGCACCUUA...
|||||||
3' GAUUGACGUGAUCUACGUGGAAU</td><td>8mer</td><td>-0.3</td><td>92</td><td>0.78</td></tr>
<tr><td>hsa-miR-144</td><td>734-740</td><td>5' ...UGGGUCAUUUUUAACUACUGUAU...
||| ||||||
3' UCAUGUAGUAGAU-AUGACAU</td><td>7mer-1A</td><td>-0.04</td><td>37</td><td>0.3</td></tr>
<tr><td></td><td>3619-3626</td><td>5' ...UCUUAGGUUUGCUAAAUACUGUA...
|||||||
3' UCAUGUAGUAGAUAUGACAU</td><td>8mer</td><td>-0.1</td><td>65</td><td>0.43</td></tr>
<tr><td>hsa-miR-101</td><td>734-740</td><td>5' ...UGGGUCAUUUUUAACUACUGUAU...
||||||
3' AAGUCAAUAGUGUCAUGACAU</td><td>7mer-1A</td><td>-0.04</td><td>30</td><td>0.3</td></tr>
<tr><td></td><td>3620-3626</td><td>5' ...CUUAGGUUUGCUAAAUACUGUAG...
||||||
3' AAGUCAAUAGUGUCAUGACAU</td><td>7mer-1A</td><td>-0.04</td><td>28</td><td>0.3</td></tr>
<tr><td>hsa-miR-23a</td><td>926-932</td><td>5' ...AAAAAAAGAAUGAGUAAUGUGAU...
|||||||
3' CCUUUAGGGACCGUUACACUA</td><td>7mer-m8</td><td>> -0.02</td><td>29</td><td>0.2</td></tr>
<tr><td>hsa-miR-23b</td><td>926-932</td><td>5' ...AAAAAAAGAAUGAGUAAUGUGAU...
|||||||
3' CCAUUAGGGACCGUUACACUA</td><td>7mer-m8</td><td>> -0.02</td><td>29</td><td>0.2</td></tr>
<tr><td>hsa-miR-23c</td><td>926-932</td><td>5' ...AAAAAAAGAAUGAGUAAUGUGAU...
|||||||
3' CCCAUUAGUGACCGUUACACUA</td><td>7mer-m8</td><td>> -0.02</td><td>28</td><td>0.2</td></tr>
<tr><td>hsa-miR-30a</td><td>955-962</td><td>5' ...GUUAUCCAGAAGUAU-UGUUUACA...
||||| |||||||
3' GAAGGUCAGCUCCUACAAAUGU</td><td>8mer</td><td>-0.1</td><td>67</td><td>0.92</td></tr>
<tr><td></td><td>3365-3371</td><td>5' ...AACAAAAUGCCUUUUUGUUUACC...
|||||||
3' GAAGGUCAGCUCCUACAAAUGU</td><td>7mer-m8</td><td>> -0.02</td><td>28</td><td>0.46</td></tr>
<tr><td>hsa-miR-30b</td><td>955-962</td><td>5' ...GUUAUCCAGAAGUAUUGUUUACA...
|||||||
3' UCGACUCACAUCCUACAAAUGU</td><td>8mer</td><td>-0.09</td><td>63</td><td>0.92</td></tr>
<tr><td></td><td>3365-3371</td><td>5' ...AACAAAAUGCCUUUUUGUUUACC...
|||||||
3' UCGACUCACAUCCUACAAAUGU</td><td>7mer-m8</td><td>-0.02</td><td>35</td><td>0.46</td></tr>
<tr><td>hsa-miR-30c</td><td>955-962</td><td>5' ...GUUAUCCAGAAGUAUUGUUUACA...
|||||||
3' CGACUCUCACAUCCUACAAAUGU</td><td>8mer</td><td>-0.09</td><td>63</td><td>0.92</td></tr>
<tr><td></td><td>3365-3371</td><td>5' ...AACAAAAUGCCUUUUUGUUUACC...
|||||||
3' CGACUCUCACAUCCUACAAAUGU</td><td>7mer-m8</td><td>-0.02</td><td>35</td><td>0.46</td></tr>
<tr><td>hsa-miR-30d</td><td>955-962</td><td>5' ...GUUAUCCAGAAGUAU-UGUUUACA...
||||| |||||||
3' GAAGGUCAGCCCCUACAAAUGU</td><td>8mer</td><td>-0.1</td><td>67</td><td>0.92</td></tr>
<tr><td></td><td>3365-3371</td><td>5' ...AACAAAAUGCCUUUUUGUUUACC...
|||||||
3' GAAGGUCAGCCCCUACAAAUGU</td><td>7mer-m8</td><td>> -0.02</td><td>28</td><td>0.46</td></tr>
<tr><td>hsa-miR-30e</td><td>955-962</td><td>5' ...GUUAUCCAGAAGUAU-UGUUUACA...
||||| |||||||
3' GAAGGUCAGUUCCUACAAAUGU</td><td>8mer</td><td>-0.1</td><td>66</td><td>0.92</td></tr>
<tr><td></td><td>3365-3371</td><td>5' ...AACAAAAUGCCUUUUUGUUUACC...
|||||||
3' GAAGGUCAGUUCCUACAAAUGU</td><td>7mer-m8</td><td>> -0.02</td><td>27</td><td>0.46</td></tr>
<tr><td>hsa-miR-761</td><td>1667-1673</td><td>5' ...ACUCAUAAAUACUAACUGCUGAU...
||||||
3' ACACAGUCAAAGUGGGACGACG</td><td>7mer-1A</td><td>-0.01</td><td>17</td><td>0.49</td></tr>
<tr><td>hsa-miR-214</td><td>1667-1673</td><td>5' ...ACUCAUAAAUACUAACUGCUGAU...
||||||
3' UGACGGACAGACACGGACGACA</td><td>7mer-1A</td><td>> -0.01</td><td>16</td><td>0.49</td></tr>
<tr><td>hsa-miR-3619-5p</td><td>1667-1673</td><td>5' ...ACUCAUAAAUACUAACUGCUGAU...
||||||
3' CGACGUGGUCGGACGGACGACU</td><td>7mer-1A</td><td>> -0.01</td><td>15</td><td>0.49</td></tr>
<tr><td>hsa-miR-215</td><td>1747-1753</td><td>5' ...CGUUGAAACGAUUUCAGGUCAAC...
||||||
3' CAGACAGUUAAGUAUCCAGUA</td><td>7mer-1A</td><td>-0.02</td><td>16</td><td>< 0.1</td></tr>
</table>

<table>
<tr><td>hsa-miR-192</td><td>1747-1753</td><td>5' ...CGUUGAAACGAUUUCAGGUCAAC...
||||||
3' CCGACAGUUAAGUAUCCAGUC</td><td>7mer-1A</td><td>-0.02</td><td>16</td><td>< 0.1</td></tr>
<tr><td>hsa-miR-200a</td><td>1787-1793</td><td>5' ...AGCAGGACUUCAGAACAGUGUUU...
|||||||
3' UGUAGCAAUGGUCUGUCACAAU</td><td>7mer-m8</td><td>-0.02</td><td>34</td><td>0.66</td></tr>
<tr><td>hsa-miR-141</td><td>1787-1793</td><td>5' ...AGCAGGACUUCAGAACAGUGUUU...
|||||||
3' GGUAGAAAUGGUCUGUCACAAU</td><td>7mer-m8</td><td>-0.02</td><td>33</td><td>0.66</td></tr>
<tr><td>hsa-miR-217</td><td>1929-1936</td><td>5' ...AUUUGUUUUACACACAUGCAGUA...
|||||||
3' AGGUUAGUCAAGGACUACGUCAU</td><td>8mer</td><td>> -0.03</td><td>6</td><td>0.41</td></tr>
<tr><td>hsa-miR-302a</td><td>1936-1942</td><td>5' ...UUACACACAUGCAGUAGCACUUU...
||||| |||||||
3' AGUGGUUUUGUACCU--UCGUGAAU</td><td>7mer-m8</td><td>-0.02</td><td>39</td><td>0.84</td></tr>
<tr><td>hsa-miR-302b</td><td>1936-1942</td><td>5' ...UUACACACAUGCAGUAGCACUUU...
||||| |||||||
3' GAUGAUUUUGUACCU--UCGUGAAU</td><td>7mer-m8</td><td>-0.02</td><td>39</td><td>0.84</td></tr>
<tr><td>hsa-miR-302c</td><td>1936-1942</td><td>5' ...UUACACACAUGCAGUAGCACUUU...
||||| |||||||
3' GGUGACUUUGUACCU--UCGUGAAU</td><td>7mer-m8</td><td>-0.02</td><td>39</td><td>0.84</td></tr>
<tr><td>hsa-miR-302d</td><td>1936-1942</td><td>5' ...UUACACACAUGCAGUAGCACUUU...
||||| |||||||
3' UGUGAGUUUGUACCU--UCGUGAAU</td><td>7mer-m8</td><td>-0.02</td><td>39</td><td>0.84</td></tr>
<tr><td>hsa-miR-302e</td><td>1936-1942</td><td>5' ...UUACACACAUGCAGUAGCACUUU...
|||| |||||||
3' UUCGUACCU--UCGUGAAU</td><td>7mer-m8</td><td>> -0.02</td><td>34</td><td>0.84</td></tr>
<tr><td>hsa-miR-372</td><td>1936-1942</td><td>5' ...UUACACACAUGCAGUAGCACUUU...
||| |||||||
3' UGCGAGUUUACAGCGUCGUGAAA</td><td>7mer-m8</td><td>> -0.02</td><td>30</td><td>0.84</td></tr>
<tr><td>hsa-miR-520a-3p</td><td>1936-1942</td><td>5' ...UUACACACAUGCAGUAGCACUUU...
|||||||
3' UGUCAGGUUUCCCUUCGUGAAA</td><td>7mer-m8</td><td>> -0.02</td><td>27</td><td>0.84</td></tr>
<tr><td>hsa-miR-520e</td><td>1936-1942</td><td>5' ...UUACACACAUGCAGUAGCACUUU...
|||||||
3' GGGAGUUUUUCCUUCGUGAAA</td><td>7mer-m8</td><td>> -0.02</td><td>25</td><td>0.84</td></tr>
<tr><td>hsa-miR-373</td><td>1936-1942</td><td>5' ...UUACACACAUGCAGUAGCACUUU...
|||||||
3' UGUGGGGUUUUAGCUUCGUGAAG</td><td>7mer-m8</td><td>> -0.02</td><td>25</td><td>0.84</td></tr>
<tr><td>hsa-miR-520b</td><td>1936-1942</td><td>5' ...UUACACACAUGCAGUAGCACUUU...
|||||||
3' GGGAGAUUUUCCUUCGUGAAA</td><td>7mer-m8</td><td>> -0.02</td><td>20</td><td>0.84</td></tr>
<tr><td>hsa-miR-520c-3p</td><td>1936-1942</td><td>5' ...UUACACACAUGCAGUAGCACUUU...
|||||||
3' UGGGAGAUUUUCCUUCGUGAAA</td><td>7mer-m8</td><td>> -0.02</td><td>20</td><td>0.84</td></tr>
<tr><td>hsa-miR-20a</td><td>1937-1943</td><td>5' ...UACACACAUGCAGUAGCACUUUG...
|||||||
3' GAUGGACGUGAUAUUCGUGAAAU</td><td>7mer-m8</td><td>> -0.02</td><td>28</td><td>0.9</td></tr>
<tr><td>hsa-miR-520d-3p</td><td>1936-1942</td><td>5' ...UUACACACAUGCAGUAGCACUUU...
|||||||
3' UGGGUGGUUUCUCUUCGUGAAA</td><td>7mer-m8</td><td>> -0.02</td><td>26</td><td>0.84</td></tr>
<tr><td>hsa-miR-106b</td><td>1937-1943</td><td>5' ...UACACACAUGCAGUAGCACUUUG...
|||||||
3' UAGACGUGACAGUCGUGAAAU</td><td>7mer-m8</td><td>> -0.02</td><td>28</td><td>0.9</td></tr>
<tr><td>hsa-miR-93</td><td>1937-1943</td><td>5' ...UACACACAUGCAGUAGCACUUUG...
|||||||
3' GAUGGACGUGCUUGUCGUGAAAC</td><td>7mer-m8</td><td>> -0.02</td><td>28</td><td>0.9</td></tr>
<tr><td>hsa-miR-20b</td><td>1937-1943</td><td>5' ...UACACACAUGCAGUAGCACUUUG...
|||||||
3' GAUGGACGUGAUACUCGUGAAAC</td><td>7mer-m8</td><td>> -0.02</td><td>28</td><td>0.9</td></tr>
<tr><td>hsa-miR-17</td><td>1937-1943</td><td>5' ...UACACACAUGCAGUAGCACUUUG...
|||||||
3' GAUGGACGUGACAUUCGUGAAAC</td><td>7mer-m8</td><td>> -0.02</td><td>27</td><td>0.9</td></tr>
<tr><td>hsa-miR-106a</td><td>1937-1943</td><td>5' ...UACACACAUGCAGUAGCACUUUG...
|||||||
3' GAUGGACGUGACAUUCGUGAAAA</td><td>7mer-m8</td><td>> -0.02</td><td>27</td><td>0.9</td></tr>
<tr><td>hsa-miR-519d</td><td>1937-1943</td><td>5' ...UACACACAUGCAGUAGCACUUUG...
|||||||
3' GUGAGAUUUCCCUCCGUGAAAC</td><td>7mer-m8</td><td>> -0.02</td><td>24</td><td>0.9</td></tr>
<tr><td>hsa-miR-135a</td><td>2688-2694</td><td>5' ...GGAGAAGUAUUGAAU---AAGCCAUU...
|||| |||||||
3' AGUGUAUCCUUAUUUUUCGGUAU</td><td>7mer-m8</td><td>-0.06</td><td>31</td><td>0.52</td></tr>
<tr><td>hsa-miR-135b</td><td>2688-2694</td><td>5' ...GGAGAAGUAUUGAAU---AAGCCAUU...
|||| |||||||
3' AGUGUAUCCUUACUUUUCGGUAU</td><td>7mer-m8</td><td>-0.06</td><td>31</td><td>0.52</td></tr>
<tr><td>hsa-miR-1</td><td>3228-3234</td><td>5' ...ACCUGUUUCUUUGGGACAUUCCG...
|||||| |||||||
3' UAUGUAUGAAGAAA---UGUAAGGU</td><td>7mer-m8</td><td>> -0.02</td><td>23</td><td>0.42</td></tr>
<tr><td>hsa-miR-206</td><td>3228-3234</td><td>5' ...ACCUGUUUCUUUGGGACAUUCCG...
||| |||||||
3' GGUGUGUGAAGGAAUGUAAGGU</td><td>7mer-m8</td><td>> -0.02</td><td>20</td><td>0.42</td></tr>
<tr><td>hsa-miR-613</td><td>3228-3234</td><td>5' ...ACCUGUUUCUUUGGGACAUUCCG...
|||||||
3' CCGUUUCUUCCUUGUAAGGA</td><td>7mer-m8</td><td>> -0.02</td><td>11</td><td>0.42</td></tr>
<tr><td>hsa-miR-184</td><td>3232-3238</td><td>5' ...GUUUCUUUGGGACAUUCCGUCCU...
|||||||
3' UGGGAAUAGUCAAGAGGCAGGU</td><td>7mer-m8</td><td>-0.22</td><td>58</td><td>0.33</td></tr>
<tr><td>hsa-miR-429</td><td>3436-3442</td><td>5' ...UGUUUUUAGCCAGGUCAGUAUUG...
||||| |||||||
3' UGCCAAAAUGGUCU--GUCAUAAU</td><td>7mer-m8</td><td>-0.02</td><td>45</td><td>0.47</td></tr>
<tr><td>hsa-miR-200c</td><td>3436-3442</td><td>5' ...UGUUUUUAGCCAGGUCAGUAUUG...
||| |||||||
3' AGGUAGUAAUGGGCC--GUCAUAAU</td><td>7mer-m8</td><td>> -0.02</td><td>38</td><td>0.47</td></tr>
<tr><td>hsa-miR-200b</td><td>3436-3442</td><td>5' ...UGUUUUUAGCCAGGUCAGUAUUG...</td><td>7mer-m8</td><td>> -0.02</td><td>38</td><td>0.47</td></tr>
</table>

<table>
<tr><td></td><td></td><td>
3'</td><td>||||| |||||||
AGUAGUAAUGGUCC-GUCAUAAU</td><td></td><td></td><td></td><td></td></tr>
<tr><td>hsa-miR-383</td><td>3450-3456</td><td>5'

3'</td><td>...UCAGUAUUGAUGAGGCUGAUCAU...
||||||
UCGGUGUUAGUGGAAGACUAGA</td><td>7mer-1A</td><td>-0.05</td><td>30</td><td>< 0.1</td></tr>
<tr><td>hsa-miR-139-5p</td><td>3621-3627</td><td>5'

3'</td><td>...UUAGGUUUGCUAAAUACUGUAGG...
|||||||
GACCUCUGUGCACGUGACAUCU</td><td>7mer-m8</td><td>-0.12</td><td>68</td><td>0.17</td></tr>
<tr><td>hsa-miR-129-5p</td><td>3765-3772</td><td>5'

3'</td><td>...AGGGGAAAUGAAUGUGCAAAAAA...
|||||||
CGUUCGGGUCUGGCGUUUUUC</td><td>8mer</td><td>-0.06</td><td>88</td><td>< 0.1</td></tr>
<tr><td>hsa-miR-4262</td><td>3758-3764</td><td>5'

3'</td><td>...UUGUUCUAGGGGAAAUGAAUGUG...
||| |||||||
GUCCAUCAGACUUACAG</td><td>7mer-m8</td><td>-0.1</td><td>72</td><td>0.77</td></tr>
<tr><td>hsa-miR-181a</td><td>3758-3764</td><td>5'

3'</td><td>...UUGUUCUAGGGGAAAUGAAUGUG...
|||||||
UGAGUGGCUGUCGCAACUUACAA</td><td>7mer-m8</td><td>-0.06</td><td>57</td><td>0.77</td></tr>
<tr><td>hsa-miR-181b</td><td>3758-3764</td><td>5'

3'</td><td>...UUGUUCUAGGGGAAAUGAAUGUG...
|||||||||
UGGGUGGCUGUCGUUACUUACAA</td><td>7mer-m8</td><td>-0.06</td><td>57</td><td>0.77</td></tr>
<tr><td>hsa-miR-181c</td><td>3758-3764</td><td>5'

3'</td><td>...UUGUUCUAGGGGAAAUGAAUGUG...
|||||||
UGAGUGGCUGUCCAACUUACAA</td><td>7mer-m8</td><td>-0.07</td><td>61</td><td>0.77</td></tr>
<tr><td>hsa-miR-181d</td><td>3758-3764</td><td>5'

3'</td><td>...UUGUUCUAGGGGAAAUGAAUGUG...
|||||||
UGGGUGGCUGUUGUUACUUACAA</td><td>7mer-m8</td><td>-0.07</td><td>61</td><td>0.77</td></tr>
</table>

Targetscan analysis - release 6.2 (June 2012) - showing only sites conserved among vertebrates

TOT miRNA = 60
