# Supplementary material for: miR-17 deregulates a core RUNX1-miRNA mechanism of CBF acute myeloid leukemia
Source: Mol Cancer. 2015 Jan 23;14:7. doi: 10.1186/s12943-014-0283-z (PMC4351976; doi:10.1186/s12943-014-0283-z)
Supplement: Additional file 3: Table S2. — List of miRNAs predicted to target KIT 3′UTR by Targetscan analysis. [file 12943_2014_283_MOESM3_ESM.pdf]

## Supplementary Table S2. MicroRNAs predicted to target KIT 3' UTR (NM_001093772 - 2158 nt) by TargetScan analysis

<table>
<thead>
<tr><th>MicroRNA</th><th>Position in KIT 3'UTR</th><th>predicted pairing of target region (top) and miRNA (bottom)</th><th>seed match</th><th>context + score</th><th>context + score percentile</th><th>PCT</th><th>Validated Kit-targeting?</th><th>Validated RUNX1-target?</th></tr>
</thead>
<tbody>
<tr><td>hsa-miR-218</td><td>844-850</td><td>5' ...AACAAUAUAACCACAAAGCACAG...<br>|||||||<br>3' UGUACCAAUCUAGUUCGUGUU</td><td>7mer-m8</td><td>-0.1</td><td>53</td><td>0.42</td><td></td><td></td></tr>
<tr><td>hsa-miR-193a-3p</td><td>1088-1095</td><td>5' ...AUGUCCUGGACACCG--GGCCAGUA...<br>|||| |||||||<br>3' UGACCCUGAAACAUCCGGUCAA</td><td>8mer</td><td>-0.22</td><td>72</td><td>0.54</td><td>YES<br>PMID:21399664</td><td>YES<br>PMID:23223432</td></tr>
<tr><td>hsa-miR-193b</td><td>1088-1095</td><td>5' ...AUGUCCUGGACACCG--GGCCAGUA...<br>|||| |||||||<br>3' UCGCCCUGAAACUCCCGGUCAA</td><td>8mer</td><td>-0.22</td><td>72</td><td>0.54</td><td>YES<br>PMID:21724256</td><td></td></tr>
<tr><td>hsa-miR-19b</td><td>1648-1654</td><td>5' ...AUCUGGAAGUAACCAUUUGCACU...<br>|||||||<br>3' AGUCAAAACGUACCUAAACGUGU</td><td>7mer-m8</td><td>-0.07</td><td>39</td><td>0.83</td><td></td><td>YES<br>PMID:17589498</td></tr>
<tr><td>hsa-miR-19a</td><td>1648-1654</td><td>5' ...AUCUGGAAGUAACCAUUUGCACU...<br>|||||||<br>3' AGUCAAAACGUAUCUAAACGUGU</td><td>7mer-m8</td><td>-0.07</td><td>39</td><td>0.83</td><td>YES<br>PMID:21880628</td><td>YES<br>PMID:17589498</td></tr>
<tr><td>hsa-miR-152</td><td>1650-1656</td><td>5' ...CUGGAAGUAACCAUUUGCACUGG...<br>|||||||<br>3' GGUUCAAGACAGUACGUGACU</td><td>7mer-m8</td><td>-0.18</td><td>72</td><td>0.72</td><td></td><td></td></tr>
<tr><td>hsa-miR-148b</td><td>1650-1656</td><td>5' ...CUGGAAGUAACCAUUUGCACUGG...<br>|||||||<br>3' UGUUUCAAGACACUACGUGACU</td><td>7mer-m8</td><td>-0.18</td><td>72</td><td>0.72</td><td></td><td></td></tr>
<tr><td>hsa-miR-148a</td><td>1650-1656</td><td>5' ...CUGGAAGUAACCAUUUGCACUGG...<br>|||||||<br>3' UGUUUCAAGACAUCACGUGACU</td><td>7mer-m8</td><td>-0.18</td><td>71</td><td>0.72</td><td></td><td></td></tr>
<tr><td>hsa-miR-4295</td><td>1649-1655</td><td>5' ...UCUGGAAGUAACCAUUUGCACUG...<br>|||| |||||||<br>3' UUCCUUUUGU----AACGUGAC</td><td>7mer-m8</td><td>-0.13</td><td>59</td><td>0.8</td><td></td><td></td></tr>
<tr><td>hsa-miR-454</td><td>1649-1655</td><td>5' ...UCUGGAAGUAACCAUUUGCACUG...<br>||| |||||||<br>3' UGGGAUAUUCGUUAU--AACGUGAU</td><td>7mer-m8</td><td>-0.13</td><td>57</td><td>0.8</td><td></td><td></td></tr>
<tr><td>hsa-miR-130a</td><td>1649-1655</td><td>5' ...UCUGGAAGUAACCAUUUGCACUG...<br>|||||||<br>3' UACGGGAAAAUUGUAACGUGAC</td><td>7mer-m8</td><td>-0.12</td><td>53</td><td>0.8</td><td></td><td></td></tr>
<tr><td>hsa-miR-301b</td><td>1649-1655</td><td>5' ...UCUGGAAGUAACCAUUUGCACUG...<br>|||||||<br>3' CGAAACUGUUAUAGUAACGUGAC</td><td>7mer-m8</td><td>-0.11</td><td>50</td><td>0.8</td><td></td><td></td></tr>
<tr><td>hsa-miR-301a</td><td>1649-1655</td><td>5' ...UCUGGAAGUAACCAUUUGCACUG...<br>|||||||<br>3' CGAAACUGUUAUGAUAACGUGAC</td><td>7mer-m8</td><td>-0.11</td><td>49</td><td>0.8</td><td></td><td></td></tr>
<tr><td>hsa-miR-130b</td><td>1649-1655</td><td>5' ...UCUGGAAGUAACCAUUUGCACUG...<br>|||||||<br>3' UACGGGAAAGUAGUAACGUGAC</td><td>7mer-m8</td><td>-0.11</td><td>49</td><td>0.8</td><td></td><td></td></tr>
<tr><td>hsa-miR-3666</td><td>1649-1655</td><td>5' ...UCUGGAAGUAACCAUUUGCACUG...<br>|||||||<br>3' AGCCGUAGAUGUGAACGUGAC</td><td>7mer-m8</td><td>-0.1</td><td>45</td><td>0.8</td><td></td><td></td></tr>
<tr><td>hsa-miR-4735-3p</td><td>1672-1678</td><td>5' ...GAGUUCUAUGCUCUCGCACCUUU...<br>|||| |||||||<br>3' UACAGAUUAAACU-CGUGGAAA</td><td>7mer-m8</td><td>-0.16</td><td>71</td><td>0.66</td><td></td><td></td></tr>
<tr><td>hsa-miR-18a</td><td>1672-1678</td><td>5' ...GAGUUCUAUGCUCUCGCACCUUU...<br>|||||||<br>3' GAUAGACGUGAUCUACGUGGAAU</td><td>7mer-m8</td><td>-0.15</td><td>64</td><td>0.66</td><td></td><td>YES<br>PMID:17589498</td></tr>
<tr><td>hsa-miR-18b</td><td>1672-1678</td><td>5' ...GAGUUCUAUGCUCUCGCACCUUU...<br>|||||||<br>3' GAUUGACGUGAUCUACGUGGAAU</td><td>7mer-m8</td><td>-0.15</td><td>64</td><td>0.66</td><td></td><td>YES<br>PMID:17589498</td></tr>
<tr><td>hsa-miR-199a-5p</td><td>1652-1658</td><td>5' ...GGAAGUAACCAUUUGCACUGGAG...<br>||||||<br>3' CUUGUCCAUCAGACUUGUGACCC</td><td>7mer-1A</td><td>-0.12</td><td>53</td><td>0.62</td><td></td><td></td></tr>
<tr><td>hsa-miR-199b-5p</td><td>1652-1658</td><td>5' ...GGAAGUAACCAUUUGCACUGGAG...<br>||||||<br>3' CUUGUCUAUCAGAUUUGUGACCC</td><td>7mer-1A</td><td>-0.11</td><td>52</td><td>0.62</td><td></td><td></td></tr>
<tr><td>hsa-miR-222</td><td>1030-1037</td><td>5' ...UUAGUUUGGAUUCUUAUGUAGCA...<br>|||||||<br>3' UGGGUCAUCGGUCUACAUCGA</td><td>8mer</td><td>-0.13</td><td>57</td><td>< 0.1</td><td>YES<br>PMID:16330772</td><td>YES<br>PMID:21076613</td></tr>
<tr><td>hsa-miR-221</td><td>1030-1037</td><td>5' ...UUAGUUUGGAUUCUUAUGUAGCA...<br>|||||||<br>3' CUUUGGGUCGUCUGUUACAUCGA</td><td>8mer</td><td>-0.11</td><td>49</td><td>< 0.1</td><td>YES<br>PMID:16330772</td><td>YES<br>PMID:21076613</td></tr>
<tr><td>hsa-miR-137</td><td>1963-1970</td><td>5' ...UAAAUAUUGAAAUGUAGCAAUAA...<br>|||| |||||||<br>3' GAUGCGCAUAAGAAU---UCGUUAUU</td><td>8mer</td><td>-0.33</td><td>97</td><td>0.92</td><td>YES<br>PMID:24412084</td><td></td></tr>
</tbody>
</table>

*Targetscan analysis - release 6.2 (June 2012) - showing only sites conserved among vertebrates*
